# TGF-β-driven LIF expression influences neutrophil extracellular traps (NETs) and contributes to peritoneal metastasis in gastric cancer

**DOI:** 10.1038/s41419-024-06594-w

**Published:** 2024-03-15

**Authors:** Fangbin Zhang, Yan Yan, Xinguang Cao, Changqing Guo, Ke Wang, Shuai Lv

**Affiliations:** 1 Department of Gastroenterology, the First Affiliated Hospital of Zhengzhou University, Zhengzhou, 450052 PR China; 2 Department of Oncology, the First Affiliated Hospital of Zhengzhou University, Zhengzhou, 450052 PR China

**Keywords:** Cancer, Diseases

## Abstract

Gastric cancer (GC), notorious for its poor prognosis, often advances to peritoneal dissemination, a crucial determinant of detrimental outcomes. This study intricately explores the role of the TGFβ-Smad-LIF axis within the tumor microenvironment in propagating peritoneal metastasis, with a specific emphasis on its molecular mechanism in instigating Neutrophil Extracellular Traps (NETs) formation and encouraging GC cellular functions. Through a blend of bioinformatics analyses, utilizing TCGA and GEO databases, and meticulous in vivo and in vitro experiments, LIF was identified as pivotally associated with GC metastasis, notably, enhancing the NETs formation through neutrophil stimulation. Mechanistically, TGF-β was substantiated to elevate LIF expression via the activation of the Smad2/3 complex, culminating in NETs formation and consequently, propelling peritoneal metastasis of GC. This revelation uncovers a novel potential therapeutic target, promising a new avenue in managing GC and mitigating its metastatic propensities.

## Introduction

Globally, gastric cancer (GC) persistently demonstrates alarming incidences and mortality, embedding a critical public health concern [1–3]. One lethal manifestation of GC, particularly peritoneal metastasis, notoriously confers detrimental patient outcomes, diminishing prognostic optimism [4, 5]. The multifaceted tumor microenvironment (TME), an intricate web encompassing a variety of cells, signaling entities, and extracellular matrix, exerts pivotal influence over GC’s initiation, progression, and subsequent metastatic ventures [6–13].

In the contemporary realm of cancer research, Neutrophil Extracellular Traps (NETs) have risen to prominence, initially identified as a neutrophil-mediated microbial defense mechanism, yet their intricate involvement in numerous diseases, notably cancer, has been increasingly underscored [14–20]. NETs are implicated in bolstering tumor proliferation, invasion, and metastasis, entwining with other pathophysiological mechanisms like inflammation and thrombosis initiation [15, 21].

Numerous signaling molecules orchestrate the formation and functionality of NETs, among which, TGF-β, a cytokine of multifaceted functionality, has been spotlighted for its intimate involvement in tumor progression and metastasis, acting notably through the activation of the Smad signaling cascade, especially the Smad2/3 complex, modulating myriad gene expressions [22–26]. Emergent research illuminates that LIF, an immunomodulatory signaling molecule, might operate as a critical effector downstream of the TGF-β/Smad2/3 pathway, furnishing a pivotal axis in tumor immunoresponsivity and potentially metastasis [27].

This inquiry, therefore, seeks to elucidate the mechanistic interplay whereby TGF-β, through the activation of the Smad2/3-LIF axis, induces NETs formation, thereby potentially galvanizing GC’s peritoneal metastasis. This exploration, we anticipate, will not only enrich the theoretical scaffold underpinning GC metastasis but may also unveil novel therapeutic avenues, bearing significant translational and clinical potential in managing this malignancy.

## Materials and methods

### Differential expression gene screening

The RNA sequencing data was obtained from the Cancer Genome Atlas database (https://cancergenome.nih.gov/), comprising raw count data from 375 gastric cancer samples and 32 non-tumor samples. The complete clinical feature data for the corresponding patients were also downloaded and extracted. Perl scripts were used to organize and extract data information. Immune-related genes were obtained from the ImmPort database (https://immport.niaid.nih.gov) and the InnateDB database (https://www.innatedb.ca) after deleting duplicate entries, totaling 2660 immune-related genes. To obtain immune genes involved in GC pathogenesis, we used the “limma” package in R (http://www.bioconductor.org/packages/release/bioc/html/limma.html) to perform differential expression analysis on GC and non-tumor samples, extracting differentially expressed genes based on |log2FC| > 1 and *P* < 0.05. Then, immune-related genes were selected from these differentially expressed genes. GSE21328 data includes high metastasis GC cell line MKN-45-p and its parent cell line MKN-45, using the R “limma” package, setting |log2FC| > 1 and *P* value < 0.05 as differential gene screening criteria, obtaining differentially expressed genes related to metastasis. The upregulated or downregulated genes differentially expressed in the screened GSE21328 and TCGA were intersected, respectively, and the obtained upregulated intersection genes were used as candidate genes. Heat maps of differentially expressed genes were also drawn using the R language.

### Functional enrichment analysis of differentially expressed genes

The candidate targets were subjected to GO enrichment analysis using the “ClusterProfiler” package in R (http://www.bioconductor.org/packages/release/bioc/html/clusterProfiler.html), including biological process (BP), molecular function (MF), and cellular component (CC) analyses, with *P* < 0.05 as the selection criterion (candidate genes were only enriched in BP). KEGG enrichment analysis was also performed through the online analysis website SangerBox (http://vip.sangerbox.com/home.html), with *P* < 0.05 as the significant enrichment screening criterion to analyze potential targets and key targets mainly affecting cell functions and signaling axes.

### Correlation between core genes and immune cell infiltration

We categorized the core genes into high and low expression groups based on the median expression and analyzed the correlation between the expression of core genes (high and low) and the clinical pathological features of gastric cancer (GC) patients using the “ComplexHeatmap” package in R. The CIBERSORT algorithm (R Script v1.03) was used to calculate the relative amounts of 22 types of immune cells in the samples, and the “corrplot” package in R was utilized to ascertain the relationships among different immune cells. The CIBERSORT R script obtained from the CIBERSORT website (https://cibersort.stanford.edu/) examined relationships between core genes and various immune cells. We analyzed the correlation between core gene expression and immune cell infiltration in GC.

### Sample collection

Our study collected tissue, peripheral blood, and ascites samples from 30 patients who underwent radical surgery and were histopathologically diagnosed with gastric cancer (GC) at our hospital. Among them, 18 patients developed peritoneal metastasis (PM group), while 12 did not (non-PM group). Additionally, samples from 5 healthy donors were collected as a control group. Before surgical removal, patients did not undergo any chemotherapy or radiation therapy. Informed consent was obtained from all patients before sample collection, and our institutional ethics committee approved the study. Venous blood from healthy donors, PM patients, non-PM patients, and ascites samples from paracentesis in PM and non-PM patients were collected following centrifugation at 2000 × *g* for 30 min. All peripheral blood and ascites samples were stored at −80 °C. Furthermore, collected tissue samples were fixed in formalin and embedded in paraffin. Table S1 shows the clinicopathological characteristics of 40 patients with GC.

### Neutrophil count in serum and ascites

Venous blood and ascites from non-PM group and PM group GC patients were analyzed for neutrophil counts in serum and ascites using the SYSMEX XNL-350 Hematology Analyzer (SYSMEX, Japan) in the clinical laboratory.

### Isolation of neutrophils and purification of NETs

Load whole blood and ascitic fluid samples from humans or nude mice into sterile vacuum containers and use Ethylenediaminetetraacetic Acid (EDTA) for high-density gradient centrifugation separation of neutrophils. Samples are layered on Ficoll-Paque PLUS (17-1440-02, GE Healthcare, USA) and centrifuged at 1000 × *g* for 30 min at room temperature. After removing the supernatant, use lysis buffer to clear red blood cells to separate neutrophils. The separated neutrophils are resuspended in RPMI 1640 medium (PM150110A, Wuhan Puno Sai Life Science Co., Ltd) containing 0.5% serum and 1% penicillin/streptomycin (P/S). Neutrophil viability is assessed using flow cytometry. The incubated neutrophils are tested in BD Fortessa FACS with Percp-conjugated mouse Ly6G antibody (46-9668-80, eBioscienceTM, ThermoFisher, USA) and APC-CD11b (17-0112-83, eBioscienceTM, ThermoFisher, USA) using FACSDiva software v6.0, with purity consistently >95% (analyzed using FlowJo software version 10.4.2). The purity of neutrophils is determined by a rapid Giemsa staining method according to the instructions in the reagent manual [28]. For NETs purification, place the neutrophil supernatant at 4 °C, centrifuge at 18,000 × *g* for 10 min, then resuspend with 100 μL cold PBS for subsequent research.

### ELISA

In brief, 5 μg/ml of anti-MPO monoclonal antibody (AF3667, R&D, USA) is coated on a 96-well microtiter plate and left overnight at 4 °C. After blocking with incubation buffer for 2 h, 4 μL of sample per well is added in accordance with the manufacturer’s instructions, combined with Quant-iTTM PicoGreenTM dsDNA (P7589, ThermoFisher, USA). The absorbance is measured at a wavelength of 405 nm using a Synergy HTX Multi-Mode Microplate Reader (BioTek, Germany). After incubating for 40 minutes at 37 °C, optical density is measured. The concentrations of TGF-β1 (ab100647, Abcam, UK) and LIF (ab242228, Abcam, UK) in serum or ascitic fluid are also quantified by ELISA according to the manufacturer’s guidelines. All values are determined through absorbance at 450 nm using a microplate reader (Epoch, BioTeK, Germany).

### Cell culture

Human gastric mucosal epithelial cells GES-1(iCell-h062), GC cells AGS(iCell-h016), and MKN-45(iCell-h345) were purchased from iCell in Shanghai, China. All cells were cultured in RPMI 1640 medium (GIBCO, USA, Catalog# 11875119) containing 10% fetal bovine serum. The culture conditions were 37 °C, 5% CO_2_, and 95% relative humidity until the cell growth density reached approximately 80%. At this point, cells were passaged.

First, neutrophils separated from designated groups were cultured in medium and incubated for 3 hours without any treatment or were exposed in vitro to either 0.25 mg/ml DNase I (Roche, Catalog# 11284932001) or GC cells (MKN-45 and AGS, at a concentration of 1 * 10^5^). Groupings were as follows: Control group (GC cells cultured with PBS), non-PM group (neutrophils from non-PM patients co-cultured with GC cells), PM group (neutrophils from PM patients co-cultured with GC cells), PM+DNase I group (DNase I-treated neutrophils from PM patients co-cultured with GC cells), GC cells group (untreated GC cells), Neutrophils(Normal) group (neutrophils from healthy subjects co-cultured with GC cells), and Neutrophils(PM)supernatant group (supernatant from culturing neutrophils from PM patients co-cultured with GC cells).

Human recombinant protein LIF(7734-LF) and anti-LIF antibody(AF-250-NA) were purchased from R&D Systems (Minnesota, USA). GC cells were treated with 20 ng/ml of rh-LIF, 10 mg/ml of anti-LIF neutralizing antibody, or a combination of both, with a treatment time of 24 h. Groupings were as follows: PM group (neutrophils from PM patients co-cultured with GC cells), PM+rh-LIF group (neutrophils from PM patients co-cultured with GC cells treated with rh-LIF), PM+rh-LIF + LIF-Ab group (neutrophils from PM patients co-cultured with GC cells treated with rh-LIF and anti-LIF).

### Construction of lentiviral vectors and cell transfection

Lentiviral overexpression vector pCDH-CMV-MCS-EF1α-copGFP (Lv-, overexpression vector, Catalog# CD511B-1, System Biosciences, USA) and lentiviral interference vector pGreenPuro(CMV) shRNA Lentivector (sh-, interference vector, Catalog# SI505A-1, System Biosciences, USA) were purchased to construct lentivirus-based LIF overexpression or LIF, Smad2, Smad3, and Smad2 + 3 (Smad2/3) silencing vectors, with silencing sequences shown in Table S2.

Using Lipofectamine 3000 reagent (L3000015, Invitrogen, New York, CA, USA), lentiviral vectors were transfected into 293T cells (CRL-3216, ATCC, USA). After 20 h, the medium was replaced with 12 mL containing 5% fetal bovine serum. Approximately 48 h later, the supernatant containing the virus was collected, filtered through a 0.45 μm cellulose acetate filter (HAWG04700, Millipore, Bedford, MA, USA), and stored at −80 °C. To construct overexpression or silenced GC cell lines, 40% confluent GC cells were incubated with the viral mixture (MOI of 20) for 8 h, and 24 h later, an additional 10 μg/mL puromycin was added to select GC cells, maintaining culture for 4 weeks to establish stable transfected cell lines. Then, RT-qPCR was used to detect the expression levels of relevant genes in each group of cells. The stably transfected GC cell lines were then co-cultured with neutrophils derived from PM, with groupings as follows: Lv-NC group (AGC cells infected with overexpression negative control lentivirus), LIF (AGC cells infected with LIF overexpression lentivirus), sh-NC (MKN-45 cells infected with interference negative control lentivirus), sh-LIF (MKN-45 cells infected with LIF interference lentivirus), TGF-β group (GC cells treated with 10 ng/mL human recombinant TGF-β1 protein (240-B, R&D Systems, MN, USA)), TGF-β +sh Smad2 group (GC cells infected with Smad2-interfering lentivirus treated with 10 ng/mL human recombinant TGF-β1 protein), TGF-β +sh Smad3 group (GC cells infected with Smad3-interfering lentivirus treated with 10 ng/mL human recombinant TGF-β1 protein), TGF-β +sh Smad2/3 group (GC cells infected with Smad2/3-interfering lentivirus treated with 10 ng/mL human recombinant TGF-β1 protein).

### Measurement of neutrophil migration in a dual-chamber system

Add 5 ×10^5^ neutrophils into the newly separated RPMI 1640 re-suspension in the upper chamber. In the lower chamber, add a mixture of RPMI 1640 and cancer cell culture medium (CM) at a 1:1 ratio, or add PM neutrophil separation medium that has been pre-treated with cancer cell CM as a chemotactic agent. Count the neutrophils migrated into the lower chamber after 3 h [29].

### ChIP-qPCR

Once GC cells have reached 70–80% confluency, 1% formaldehyde is added, and cells are fixed at room temperature for 10 min to enable intra-cellular DNA and proteins to be cross-linked. After the cross-linking, the material is subjected to sonication, being sheared for 10 s at a time, with a 10-s interval, repeated 15 times, ensuring the breaking into fragments of an appropriate size. The solution is centrifuged at 12,000 × *g* at 4 °C, and the supernatant is collected and then divided into two tubes. Negative control antibody rabbit anti-IgG (1:100, ab109489, Abcam, UK) and target protein-specific antibody anti-Smad2/3 (ab202445, 1:100, Abcam, UK) are added, respectively, and an overnight incubation at 4 °C is performed. Protein Agarose/Sepharose is used to precipitate endogenous DNA-protein complexes, and after brief centrifugation to remove the supernatant, non-specific complexes are washed away, cross-links are reversed with an overnight incubation at 65 °C, and DNA fragments are recovered using phenol/chloroform extraction. Qualitative analysis is conducted via 3% agarose gel electrophoresis, and the sequences of the ChIP-qPCR products are presented in Table S3.

### Dual-luciferase reporter assay

293T cells (CRL-3216, ATCC, USA) are cultured in a 48-well plate for 24 hours. The pGL3-promoter luciferase reporter plasmid (E1761, Promega, Promab Biotechnologies, Beijing, China) is utilized to construct LIF wild type (WT) or mutant (Mut) plasmids (with the mutation site as “GCCCAGACA”). These are co-transfected into 293T cells with sh-Smad2/3 or negative control (shNC) plasmids for 48 h. The Pierce™ Renilla-Luciferase Dual Assay Kit (16186, Thermo Fisher Scientific China Co., Ltd) is employed to detect Renilla luciferase (Rluc) and firefly luciferase (Luc) fluorescence, using Renilla luciferase as an internal reference. The relative luciferase activity is determined by the ratio of firefly luciferase (Luc) to Renilla luciferase (Rluc). The experiment is repeated three times.

### RT-qPCR

According to the instructions, total RNA is extracted using Trizol reagent (15596026, Invitrogen, USA), and RNA is reverse-transcribed to cDNA following the PrimeScript RT reagent Kit manual (RR047A, Takara, Japan). The synthesized cDNA is subjected to RT-qPCR analysis using the Fast SYBR Green PCR Kit (11736059, Thermo Fisher Scientific China Co., Ltd, Shanghai, China), with three replicates set for each well. GAPDH is used as an internal reference. Relative expression is calculated using the 2-ΔΔCt method. The experiment is repeated three times. The primer sequences used for RT-qPCR in our research are shown in Table S4, and Takara synthesized the primer sequences.

### Western blot

Cellular protein samples are isolated from tissue and whole-cell lysates and quantified using the Pierce BCA Protein Assay Kit (23227, Thermo Fisher, USA). Tissue and cell proteins are extracted using RIPA buffer. Twenty micrograms of protein from each sample is loaded onto SDS-PAGE gels and transferred to nitrocellulose membranes. After blocking with 5% non-fat milk for 1 h, incubation is performed using the following antibodies: mouse anti-GAPDH (ab8245, Abcam, Cambridge, UK), Smad2/3 (ab202445, 1:100, Abcam, Cambridge, UK), LIF (sc-515931, 1:100, Santa Cruz Biotechnology Co., Ltd, China). All antibodies are diluted according to the instructions. On the following day, imaging is captured using an enhanced chemiluminescence reagent (WP20005, Thermo Fisher, USA) and a ChemiDoc XRS Plus luminescent image analyzer (Bio-Rad Laboratories, California, Hercules, USA). The Western blot images are quantitatively analyzed for grayscale values of bands from each group using Image J analysis software, with GAPDH as an internal reference. The experiment is repeated three times. All Original western blots images can be found in the Supplementary files.

### CCK-8 experiment

Cell proliferation was assessed using a CCK-8 assay kit (40203ES60, Yeasen, Shanghai, China). Cells in the logarithmic growth phase were harvested and adjusted to a concentration of 5 × 10^4^ cells/mL with complete culture medium, then seeded into a 96-well culture plate, with 100 μL of cell culture medium added per well, and incubated for 0, 24, 48, and 72 h. The supernatant was quickly discarded and replaced with fresh culture medium, followed by adding 10 μL of CCK-8 solution per well and further incubating at 37 °C for 2 h. The absorbance value (A) was measured using a Multiskan FC Microplate Photometer (51119080, purchased from Thermo Fisher Scientific, USA) with a detection wavelength of 450 nm. Three parallel wells were set up for each group, and the mean value was taken. The experiment was repeated three times.

### Transwell experiment

Migration and invasion assays were conducted using 24-well plates with 8 μm Transwell chambers (3422, Corning, USA). For the invasion assay, 100 μL of matrix gel was spread in each chamber and incubated at 37 °C for 2 hours. GC cells were digested, washed twice with PBS, resuspended in serum-free culture medium, and adjusted to a cell density of 3 × 10^5^ cells/mL. Three chambers were set up for each group, with 200 μL of cell suspension added. The lower chamber was filled with 700 μL of complete culture medium and placed in a 37 °C, 5% CO_2_ incubator. After incubating for 48 h, the chambers were fixed with methanol for 30 minutes and stained for 5 minutes in 0.05% crystal violet (G1062, Solarbio, Beijing, China, https://www.solarbio.com/). Cells inside the chamber were wiped off using a cotton swab, followed by observation and photography under an inverted microscope (IX73, OLYMPUS, Japan, https://www.olympus-lifescience.com.cn). ImageJ software was used for image processing and quantification. For the migration assay, spreading matrix gel in the chambers was unnecessary; all other experimental steps were the same as for the invasion assay. The experiment was repeated three times.

### Immunofluorescence co-staining

Firstly, tissues were fixed with 4% paraformaldehyde at 4 °C, washed twice with PBS, then embedded in paraffin and sectioned into 5-μm-thick slices. Next, paraffin-embedded tissue sections underwent deparaffinization, rehydration, and antigen retrieval in EDTA buffer. The slices were blocked using an Fc receptor blocker (CDN-ZF1, Beijing Anbace Biotechnology Co., Ltd, https://www.abace-biology.com/) and subsequently with 5% bovine serum albumin at room temperature for 25 min.

In various treatment groups, neutrophils were fixed in 4% paraformaldehyde overlaid on a layer of poly-L-lysine (P3543, Sigma-Aldrich LLC.) at room temperature for 15 min. Additionally, 50 nM Phorbol-12-myristate-13-acetate (PMA, P1585, Sigma-Aldrich) was used to stimulate them at 37 °C and 5% CO_2_ for 4 h to induce NETs as a positive control.

GC cells were fixed with 4% paraformaldehyde at room temperature for 15 min, followed by two PBS washes. Then, cells were permeabilized with 0.5% Triton X-100 (P0096, Beyotime) for 10 min. Subsequently, cells were incubated overnight at 4 °C with the following antibodies: anti-Cit-H3 (1:100, ab5103, Abcam, UK), anti-MPO (10 μg/ml, AF3667, R&D, USA), anti-LIF (1:100, sc-515931, Santa Cruz Biotechnology (Shanghai) Co., Ltd, China), anti-E-cadherin (1:400, ab231303, Abcam, UK), anti-Vimentin (1:500, ab92547, Abcam, UK), anti-N-Cadherin antibody (1:200, ab18203, Abcam, UK), anti-Snail antibody [CL3700] - N-terminal (1:200, ab224731, Abcam, UK), and anti-Twist antibody [10E4E6] (1:200, ab175430, Abcam, UK). Subsequently, cells were incubated with the fluorescently labeled secondary antibodies for 30 min, followed by two PBS washes. Cells were counterstained with DAPI (2 μg/mL, D3571, Thermo Fisher, USA) or Hochest 33258 (1 g/mL, H1398, Thermo Fisher, USA) and imaged using a fluorescence microscope (ECLIPSE E800, Nikon, Japan, http://nikon.com.cn/sc_CN/). Finally, image analysis was performed using Photoshop 5.0 software.

### Construct a PM nude mouse model

We purchased 50 BALB/c nude mice (8 weeks old, 20–25 g) from Beijing Vital River Laboratory Animal Technology Co., Ltd (Production batch number: SCXK-2021-0011, Beijing, China). All experimental procedures were approved by our Institutional Animal Care and Use Committee. GC PM model nude mice were divided into 5 groups, each consisting of 10 mice. Initially, MKN-45 cells, containing stably transduced shNC or shLIF lentivirus, were cultured in a 10 cm dish and then mixed with PM group neutrophils at a 1:1 ratio, co-culturing for 3 days (medium changed every 8 h). Afterward, more than 5 × 10^6^ GC cells mixed with neutrophils were injected intraperitoneally into the PM model under total anesthesia. After injecting 200 μL of a solution containing 150 mg d-luciferin (L9504, Sigma-Aldrich LLC., USA), metastatic progression was monitored and quantified using the in vivo imaging system Spectrum (Caliper Life Sciences, Waltham, MA, USA) for 20 days. Upon detection of the luciferase signal, all mice were euthanized post-CO_2_ anesthesia, followed by ELISA, H&E staining, or immunofluorescence analysis of blood or peritoneal samples.

The grouping information for the PM model experiment is as follows: MKN-45 group (mice injected with MKN-45 cells mixed with PM neutrophils). DNase I group (mice injected with MKN-45 cells mixed with PM neutrophils treated with DNase I). sh-NC group (mice injected with stably transduced MKN-45 cells mixed with PM neutrophils). Sh-LIF group (mice injected with stably transduced MKN-45 cells mixed with PM neutrophils). sh-NC + TGF-β group (mice injected with sh-NC stably transduced MKN-45 cells mixed with PM neutrophils, followed by 300 μg/mouse injection of TGF-β1). sh-LIF + TGF-β group (mice injected with sh-LIF stably transduced MKN-45 cells mixed with PM neutrophils, followed by 300 μg/mouse injection of TGF-β1). Subsequent intraperitoneal TGF-β1 treatments were administered every 5 days, continuing for 20 days.

### H&E staining

Utilizing the Hematoxylin and Eosin (H&E) staining kit (C0105, Beyotime, Beyotime,https://www.beyotime.com/), staining was performed as follows: Initially, omental tissue was fixed in 10% neutral-buffered formalin at 4 °C for 24 h. Subsequently, dehydration, wax immersion, embedding, and sectioning were carried out. Sections were routinely deparaffinized with xylene and were subjected to gradient alcohol hydration and distilled water washing. Thereafter, sections were placed in a hematoxylin staining solution and were stained for 5–10 min. Excess stain was washed away with deionized water for approximately 10 min, followed by eosin staining for 30 s to 2 min. Gradient alcohol dehydration was then performed, followed by clarification using xylene. Lastly, neutral balsam or another sealing agent was used for sealing, and observations and photographs were taken under an inverted microscope (IX73, acquired from OLYMPUS, Japan, https://www.olympus-lifescience.com.cn).

### Statistical analysis

Our research data was analyzed using the SPSS software package (Version 23.0, IBM SPSS) or GraphPad Prism software (Version 8.0). Quantitative data are presented as mean ± standard deviation. Normality and homogeneity of variance were first tested. For data that was normally distributed and had homogeneity of variance, an unpaired t-test was utilized for intergroup comparison, while one-way analysis of variance (ANOVA) or ANOVA for repeated measures was employed for multiple-group comparisons. Pearson’s method was used to analyze the correlation between two indices. A *P*-value < 0.05 was considered to indicate statistical significance.

## Results

### GC PM could promote neutrophil recruitment and NETs formation

**Fig. 1:**
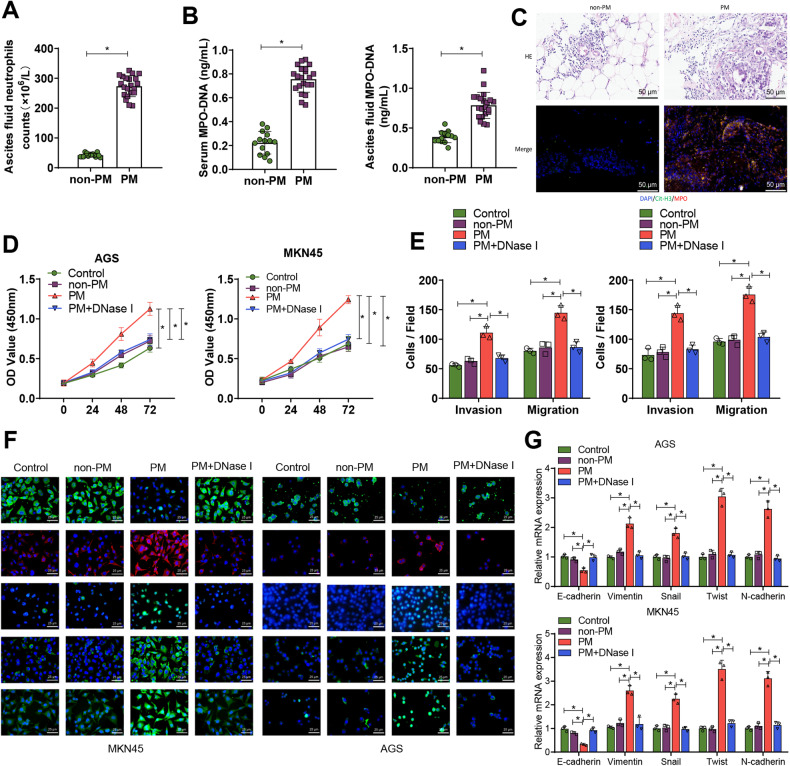
Effects of neutrophil NET formation on GC cell proliferation, invasion, migration, and EMT. **A** Neutrophil counts in ascites were determined in the non-PM group (*n* = 12) and PM group (*n* = 18); **B** ELISA evaluated levels of MPO-DNA complexes in serum and ascites in the non-PM group (*n* = 12) and PM group (*n* = 18) to assess NETs levels; **C** Representative images of H&E and immunofluorescence staining of Cit-H3.MPO in omental tissues from non-PM group (*n* = 12) and PM group (*n* = 18), with blue indicating cell nuclei, green indicating Cit-H3, and red indicating MPO; **D** Proliferation ability of GC cells was detected by CCK-8 assay after co-culturing with neutrophils from each group; **E** Transwell assay was used to assess the migration and invasion ability of GC cells after co-culturing; **F** Immunofluorescence was used to detect the expression of EMT-related factors. E-cadherin and Snail were labeled in red, while Vimentin, Twist, and N-cadherin were labeled in green. The cell nuclei were labeled in blue. **G** RT-qPCR was performed to detect the expression of E-cadherin, Vimentin, Snail, Twist, and N-cadherin in GC cells after co-culture. *P < 0.05, cell experiments were repeated at least three times.

GC PM (Gastric Cancer Peritoneal Metastasis) can promote the recruitment of neutrophils and the formation of NETs (Neutrophil Extracellular Traps). When the peritoneum is stimulated by antigens, neutrophils can enter the peritoneal cavity through high endothelial venules (HEVs) in the omental milky spots, releasing cytokines, chemokines, and granule proteins to create a microenvironment conducive to tumor growth while also inducing tumor cell metastasis [29, 30]. In our study, we first assessed the content of neutrophils in the ascites of GC patients. The results showed a significant increase in the number of neutrophils in the PM group compared to the non-PM group (Fig. 1A). Neutrophils generally promote cancer metastasis by forming NETs structures, which release granule proteins and chromatin [31, 32]. To determine whether GC PM could stimulate neutrophils to release NETs, we utilized ELISA to evaluate NETs levels by measuring the MPO-DNA complexes in serum and ascites. The results indicated that levels of NETs in the ascites and serum of GC patients with PM were significantly higher than those in GC patients without PM (Fig. 1B).

Furthermore, H&E staining revealed that after PM, there is substantial inflammatory cell infiltration in the omental tissue, and the metastatic nodules are also significantly increased. Immunofluorescence co-staining for citrullinated histone-3 (Cit - H3) and myeloperoxidase (MPO) in omental tissue showed that levels of Cit - H3 and MPO in the omental tissue of GC patients with PM were significantly higher than those in non-PM (Fig. 1C).

We further isolated neutrophils from the ascites of both the non-PM and PM groups and flow cytometry verified that the purity of the separated neutrophils exceeded 90% (Fig. S1A). The formation of NETs was observed through immunofluorescence co-staining. The results showed that levels of Cit-H3 and MPO in the isolated neutrophils from the PM group were significantly elevated compared to the non-PM group. Moreover, when DNase I was added to specifically block NETs, the aforementioned enhanced effect could be significantly eliminated (Fig. S1B).

Subsequently, we focused our research on whether NETs, induced by PM in neutrophils, facilitate the proliferation and metastasis of GC cells. After co-culturing MKN-45 and AGS cells with neutrophils, we noticed that compared to the control and non-PM groups, neutrophils from the PM group promoted the proliferation, invasion, migration, and EMT of GC cells. However, when DNase I was added to obstruct NETs, the aforementioned enhanced effect could be significantly abolished (Fig. S1C and Fig. 1D–G).

Furthermore, we found that in the supernatant of the culture medium of neutrophils derived from the serum of the normal group, non-PM group ascites, and PM group ascites, MPO-DNA was not detected (Fig. S2A). Additionally, the supernatant from neutrophils of healthy donor serum and PM group neutrophils did not affect the proliferation, invasion, and migration of GC cells (Fig. S2B–D). Simultaneously, immunofluorescence co-staining results showed that mere co-culturing of normal neutrophils with GC cells could not stimulate neutrophils to release NETs (Fig. S2E).

### LIF may be a key target for neutrophil infiltration in the tumor microenvironment that mediates peritoneal metastasis of gastric cancer

To further explore the critical target mediating neutrophil infiltration in the tumor microenvironment during GC peritoneal metastasis, we initially intersected TCGA GC immune-related differential genes with differential genes between the high metastatic GC cell line MKN-45-P and its parental cell line MKN-45 in the GEO database chip GSE21328. A total of 13 upregulated intersected genes and 2 downregulated intersected genes were obtained (Fig. S3A, B). Among them, the 13 upregulated intersected genes were differentially expressed in the TCGA and GSE21328 chip (Fig. S3C).

We performed KEGG and GO function enrichment analysis on these 13 upregulated intersected genes to probe the key target of neutrophil infiltration in the GC tumor microenvironment. We discovered that they were mainly enriched in pathways and related functional pathways like Cytokine-cytokine receptor interaction, TGF-beta signaling pathway, and multi-multicellular organism process involving INHBE, TNFSF11, LIF, CXCL11, STC2, and IDO1 (Fig. S4A, B, Table S5), indicating that these 13 candidate targets might be involved in cell-to-cell communication within the GC tumor microenvironment.

**Fig. 2:**
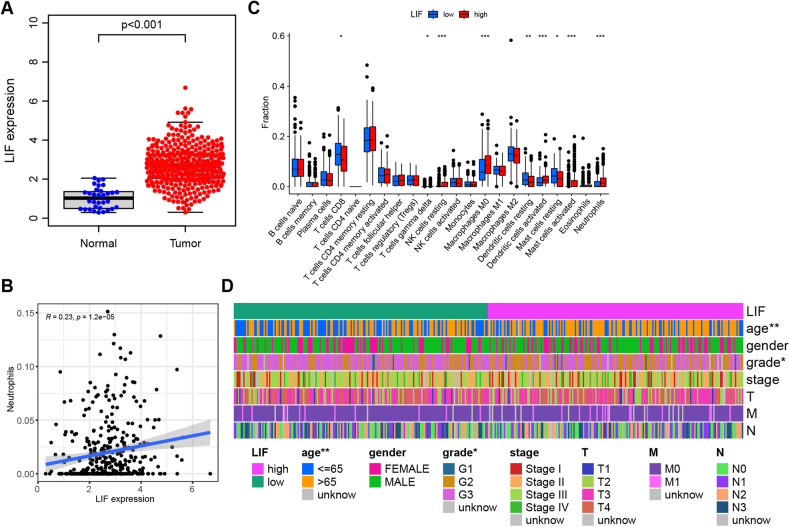
Key targets for tumor microenvironment neutrophil infiltration mediated by bioinformatics screening of GC peritoneal metastasis. **A** Differential expression of LIF in the TCGA database: Normal, *n* = 37; Tumor, *n* = 375; **B** Correlation analysis of LIF and neutrophil infiltration, *n* = 375; **C** Analysis of differences in immune cell content between high expression group (red) and low expression group (green) of LIF; **D** Clinical correlation analysis of high and low expression of LIF. *P < 0.05, **P < 0.01, ***P < 0.001.

Further, utilizing CIBERSORT to analyze the correlation between immune cell infiltration and key genes in GC patients in the TCGA database, results showed that genes positively correlated with neutrophils included LIF, ULBP2, TRIB3, and IFI30 (Fig. 2B and Fig. S5). Combining the above analysis results and considering existing research indicating that LIF is associated with GC peritoneal metastasis [33], we selected LIF as the object of subsequent research.

As shown in Fig. 2A, LIF displays differential expression in GC samples in the TCGA database. We further divided LIF into high and low expression groups according to the median expression level of LIF, and through analyzing the differences of 22 immune cells between the LIF high expression group and low expression group, we found that LIF gene expression was significantly correlated with numerous immune cells, including neutrophils (Fig. 2C). Additionally, we also found that the levels of LIF were significantly correlated with age and tumor grade staging (Fig. 2D). Additionally, we observed a significant correlation between neutrophil infiltration and age, tumor stage, and T classification, further highlighting the role of neutrophils in gastric cancer metastasis (Fig. S6).

### LIF could induce the recruitment of neutrophils and the formation of NETs in the tumor microenvironment of GC

**Fig. 3:**
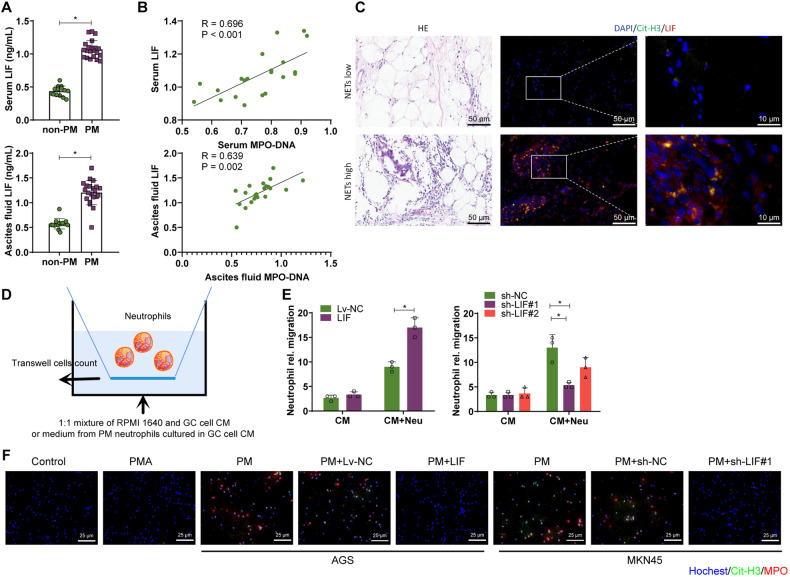
Influence of LIF on neutrophil recruitment and formation of NETs in the GC tumor microenvironment. **A** Levels of LIF in serum and ascites of both non-PM group (*n* = 12) and PM group (*n* = 18) GC patients; **B** Pearson analysis of the correlation between serum MPO-DNA and LIF levels in PM group (*n* = 18) GC patients, and the correlation between ascites MPO-DNA and LIF levels; **C** Representative H&E and immunofluorescence co-staining images of Cit-H3 and LIF in the greater omentum tissue of PM group GC patients, with Hochest staining for nuclei in blue, Cit-H3 staining in green, and LIF staining in red; **D** Schematic diagram of the recruitment of neutrophils by CM from AGC cells or MKN-45 cells overexpressing or interfering with LIF, or by CM from neutral neutrophils (Neu) pretreated with CM from these cancer cells for 12 h; **E** Neutrophil counts in each group of migration; **F** Immunofluorescence co-staining detection of Cit-H3 and MPO levels, with Hochest staining for nuclei in blue, Cit-H3 staining in green, and MPO staining in red. *P < 0.05, cell experiments were repeated at least three times.

To determine the correlation between LIF levels and NETs formation, we first tested the LIF levels in gastric cancer patients’ serum and ascites. As shown in Fig. 3A, the levels of LIF in the serum and ascites of the PM group were higher than those in the non-PM group. The correlation analysis showed a significant positive correlation between the levels of LIF in serum and ascites and the corresponding levels of MPO-DNA (Fig. 3B). In addition, the immunofluorescence co-staining of Cit-H3 and LIF was performed on GC patients with PM retinal tissues. The results showed a positive correlation between LIF expression and NETs release (Fig. 3C).

Next, we discussed the impact of LIF on neutrophil recruitment. First, we compared the expression levels of LIF in normal human gastric mucosal epithelial cells and GC cells. The results showed that the levels of LIF in AGC cells and MKN-45 cells increased compared to the GES-1 group, with MKN-45 cells having higher levels of LIF (Fig. S7A). We further used lentiviral vectors to overexpress or knockdown LIF in AGC cells or MKN-45 cells and detected the expression levels of LIF mRNA using RT-qPCR and ELISA. Results showed that compared with the Lv-NC group, the expression of LIF mRNA in LIF group AGC cells increased, and the LIF protein content in AGC cell culture medium (CM) also increased, while the opposite trend was observed after interfering with LIF in MKN-45 cells (Fig. S7B, C).

We found overexpression and interference with LIF in glomerular cells did not directly attract neutrophils in the conditioned medium (CM) (Fig. 3D, E). However, when CM treated with slow virus handling GC cells expressing excessive or interfering LIF was used to pretreat the PM neutrophils, it was found that compared to the corresponding control group, the LIF group was able to attract more neutrophils, while the sh-LIF group recruited fewer neutrophils (Fig. 3D, E).

In addition, we found through immunofluorescence co-staining that PM neutrophils cultured in LIF-treated GCCM were able to promote the formation of neutrophil extracellular traps (NETs) structures, which were similar to the structures formed by the NETs inducer, PMA, compared to the control group. On the contrary, sh-LIF reduced the formation of NETs by CM stimulation (Fig. 3F). In conclusion, LIF could promote the recruitment of neutrophils and the formation of NETs in the tumor microenvironment of GC.

### NETs induce proliferation, invasion, migration, and EMT in GC cells

**Fig. 4:**
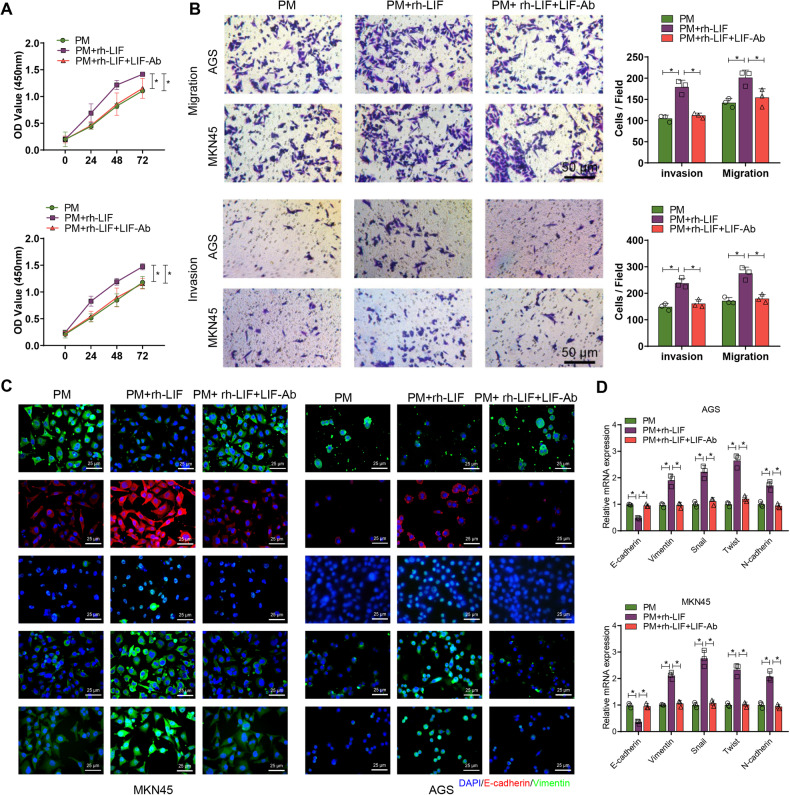
LIF activation affects GC cells’ proliferation, invasion, migration, and epithelial-mesenchymal transition (EMT) when co-cultured with neutrophils. **A** After inducing co-cultivation of GC cells from PM with neutrophils, which were pretreated with 20 ng/ml recombinant LIF protein and 10 mg/mL neutralizing antibody against LIF, the proliferation ability of GC cells was assessed using the CCK-8 assay; **B** The migration and invasion ability of GC cells after co-cultivation was assessed using the Transwell assay; **C** Immunofluorescence staining to detect the expression of EMT-related factors. E-cadherin and Snail were labeled in red, while Vimentin, Twist, and N-cadherin were labeled in green. The cell nucleus was labeled in blue. **D** RT-qPCR to detect the expression of E-cadherin, Vimentin, Snail, Twist, and N-cadherin in GC cells after co-culture. *P < 0.05, cell experiments were repeated at least three times.

To further determine the role of LIF in NETs-mediated EMT as well as invasion, migration, and proliferation, we co-cultured GC cells induced with recombinant LIF protein and anti-LIF neutralizing antibody with neutrophils from PM. The results showed that compared with GC cells co-cultured only with PM neutrophils, the GC cells in the rh-LIF group demonstrated significantly increased proliferation, invasion, migration, and EMT. Meanwhile, LIF-Ab was able to reverse the effects of recombinant LIF protein in promoting proliferation, invasion, migration, and EMT in GC cells co-cultured with neutrophils (Fig. 4A–D).

### TGF-β induces the expression of LIF by activating the Smad2/3 complex

**Fig. 5:**
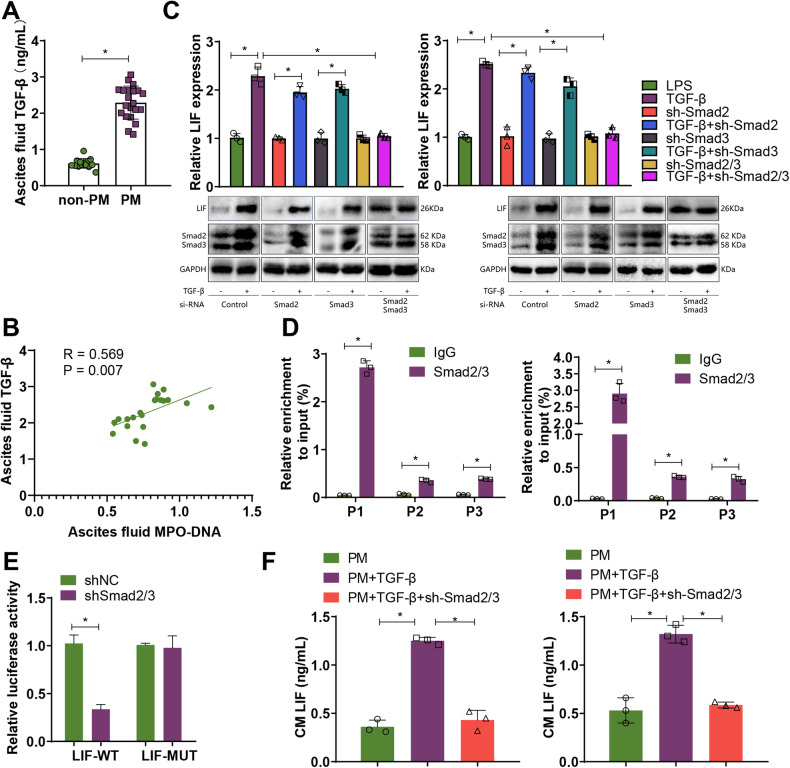
Transcriptional regulation of LIF expression by the TGF-β/Smad signaling axis. **A** Levels of TGF-β in ascites of non-PM group (*n* = 12) and PM group (*n* = 18) GC patients; **B** Pearson analysis of the correlation between MPO-DNA in ascites of PM group (*n* = 18) GC patients and TGF-β levels; **C** Detection of LIF mRNA levels by RT-qPCR and protein expression of LIF, Smad2/3 by Western Blot; **D** ChIP-qPCR to detect potential binding sites of Smad2/3 in the LIF promoter region; **E** Dual-Luciferase reporter assay to investigate the transcriptional regulation of LIF by Smad2/3; **F** ELISA to measure the levels of LIF in GC cell culture medium. * Indicates that *P* < 0.05 and cell experiments are repeated at least three times.

Based on prior KEGG and GO enrichment analysis, we found that LIF can be enriched in the TGF-β signaling pathway and SMAD protein signal transduction. Existing research indicates that LIF expression depends on the activation of the TGF-β/Smad complex [34, 35]. We also observed that, in clinical GC tissues, LIF is positively correlated with TGF-β, Smad2, and Smad3 (Fig. S8A). Moreover, TGF-β levels in peritoneal fluid of GC patients in the PM group were significantly higher than those in the non-PM group, and peritoneal fluid TGF-β levels were positively correlated with MPO-DNA levels (Fig. 5A, B).

To demonstrate the involvement of Smads in TGF-β-induced LIF expression, we individually and jointly interfered with Smad2 and Smad3. Our experimental results indicated that compared to the control group, TGF-β can induce an increase in LIF levels, while single knockdowns of Smad2 or Smad3 did not significantly inhibit the increase of LIF levels induced by TGF-β. However, when Smad2 and Smad3 were both knocked down, TGF-β-induced LIF levels significantly decreased, indicating that the expression of TGF-β-induced LIF requires the participation of the Smad2/3 complex (Fig. 5C).

In addition, the JASPAR database shows the presence of binding sites in the LIF promoter region. To further investigate whether Smad2/3 is involved in the transcriptional regulation of LIF, we selected three putative Smad2/3 binding sites (P1–P3, Fig. S8B, C) in the LIF promoter using JASPAR. ChIP-qPCR results showed that Smad2/3 was highly enriched in the P1 region of the LIF promoter (Fig. 5D). We further mutated the P1 region site and studied the relationship between Smad2/3 and LIF through a dual-luciferase reporter assay. Results showed that upon knockdown of Smad2/3, LIF-WT luciferase activity significantly decreased, while LIF-MUT showed no significant change (Fig. 5E).

Further using TGF-β1 (a TGF-β agonist) and sh Smad2/3, we determined the role of the TGF-β/Smad signaling axis in LIF-mediated GC cell proliferation, invasion, and migration. Results demonstrated that after co-culturing with the PM + TGF-β group, compared to the PM group, LIF levels in GC cell culture medium significantly increased, and GC cell proliferation, invasion, and migration also significantly increased, while sh-Smad2/3 could reverse the promotional effect of TGF-β on GC cells (Fig. 5F, Fig. S9). The evidence suggests that TGF-β can upregulate the expression of LIF by activating the Smad2/3 complex.

### Interfering with LIF expression could inhibit peritoneal metastasis of GC induced by NETs in the body

**Fig. 6:**
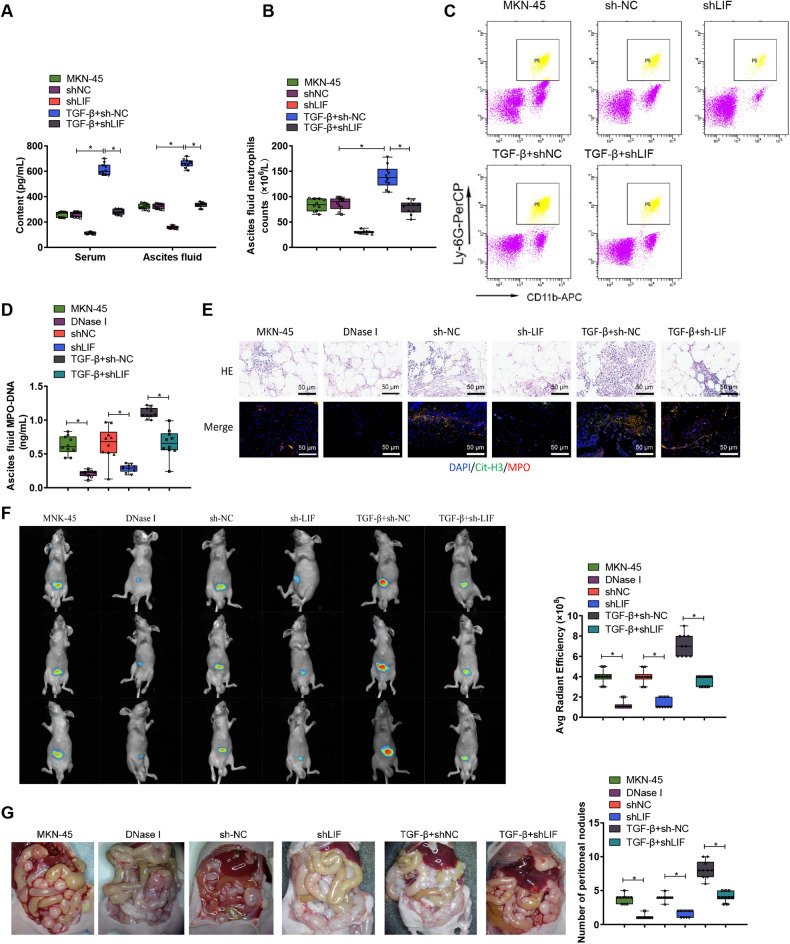
The in vivo induction of PM by NETs may be associated with the activation of LIF. **A** ELISA was used to measure the levels of LIF in serum and ascites of nude mice; **B** The neutrophil count in ascites of the PM nude mouse model was determined; **C** Flow cytometry was performed to analyze the proportion of neutrophils in ascites of nude mice; **D** ELISA was conducted to assess the levels of MPO-DNA complexes in the ascites of nude mice, indicating the levels of NETs; **E** Representative images of H&E and immunofluorescence staining of Cit-H3.MPO in the retinal tissue of nude mice, with cell nuclei labeled in blue, Cit-H3 in green, and MPO in red, scale bar = 50 μm; **F** Representative images and quantification of peritoneal metastatic lesions in PM nude mice. **G** Observation and statistical analysis of visible metastatic nodules on the mesentery of nude mice. *N* = 10.

To validate this finding, we injected MKN-45 cells infected with a lentivirus carrying shLIF into a nude mouse model to establish peritoneal metastasis (PM). The results showed that, compared to the group injected only with MNK-45 cells or the group injected with stable shNC, the levels of LIF in the serum and peritoneal fluid of the shLIF group were significantly reduced. Whereas, LIF levels in the serum and peritoneal fluid of the TGF-β+sh-NC group were significantly increased, but after injecting TGF-β into the shLIF group, the promotional effect of TGF-β on LIF was inhibited (Fig. 6A).

Further investigation into the recruitment of neutrophils showed that compared to the MKN-45 group or sh-NC group, the number and proportion of CD11b + Ly6G+ neutrophils in the peritoneal metastatic tumors of the shLIF group were significantly reduced. The neutrophil count and ratio in the TGF-β+shLIF group remained essentially the same, and compared to the TGF-β+sh-NC group, the neutrophil number and proportion in the TGF-β+shLIF group were significantly reduced (Fig. 6B, C).

To verify the role of NETs in the PM nude mouse model, we further injected DNase I to inhibit the formation of NETs in the body. The results showed that compared to the MKN-45 group or shNC group, the MPO-DNA content in the peritoneal fluid of both the DNase I and sh-LIF groups was significantly reduced (Fig. 6D). Immunofluorescence co-staining showed that the expression of Cit - H3 and MPO in both the DNase I and sh-LIF groups was also significantly reduced, and interfering with LIF could inhibit the promotional effect of TGF-β on NETs (Fig. 6D, E). These results indicate that, within PM nude mice, LIF activated through TGF-β can inhibit the formation of NETs. Further examination of the effect of LIF on peritoneal metastasis showed that, compared to the MNK-45 group or shNC group, both the DNase I and sh-LIF groups inhibited the ability of the nude mice to develop peritoneal metastasis, and interfering with LIF could inhibit the promotional effect of TGF-β on PM (Fig. 6F, G).

## Discussion

**Fig. 7:**
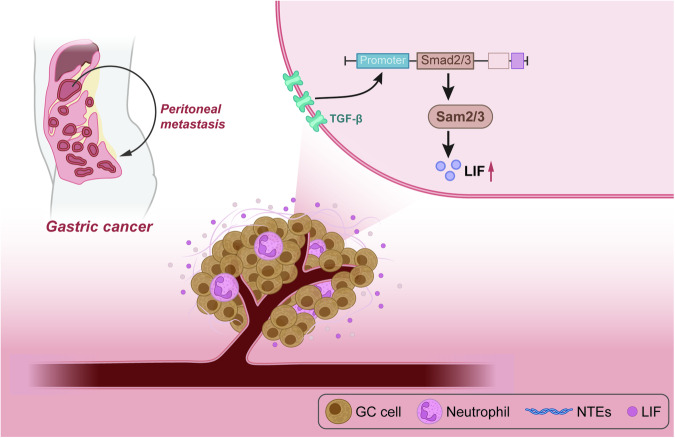
Schematic diagram of the molecular mechanism by which the TGF-β-Smad-LIF axis in the tumor microenvironment promotes the formation of neutrophil extracellular traps (NETs) and facilitates peritoneal metastasis of gastric cancer.

Navigating through the nuanced cellular machinations within the tumor microenvironment (TME), this study unearthed pivotal insights elucidating TGF-β’s capability to augment LIF expression, activated via the Smad2/3 complex. This cascade subsequently incites neutrophil recruitment and NETs formation, ultimately charting a course towards the peritoneal metastasis of gastric cancer (Fig. 7).

Renowned as a principal culprit in digestive system tumor mortalities, gastric cancer (GC) perpetuates significantly poor prognoses, prominently as a consequence of its metastatic proclivities [1]. Even though prior investigations have demystified myriad metastasis mechanisms within GC, a dense fog still shrouds numerous facets of its molecular pathways [1, 36–38]. The robust, complex pathways traversed by GC during metastasis, albeit subject to extensive research, continue to harbor unexplored molecular mechanisms. This investigation, therefore, introduces a fresh theoretical framework underpinning the metastatic mechanics of GC.

TGF-β, recognized for its regulatory virtuosity across numerous tumor varieties and functionally diverse roles, oscillates between inhibiting and propelling tumor progression dependent upon the specific neoplastic context [39–41]. This research pioneers the identification of TGF-β as a linchpin in the peritoneal metastasis of GC, particularly via its stewardship of the Smad2/3-LIF axis.

NETs have previously been spotlighted for their cardinal role across various tumors, with a particular focus on orchestrating tumor immune microenvironments [25, 42, 43]. Their association with tumor proliferation, metastasis, and immune evasion has been substantively documented [44–46]. This investigation diverges slightly from extant viewpoints, spotlighting NETs as determinative players in GC’s peritoneal metastasis.

Concurrently, LIF, an entity influential across various physiological and pathological scenarios—especially in mediating inflammatory responses—has previously been indicted as a pro-tumor agent in certain neoplasms [25, 47]. This research venture sheds light on a profound synergistic interplay between LIF and TGF-β, revealing their orchestrated role in the metastatic journey of GC through TGF-β‘s mediation.

Historically, TGF-β, LIF, and NETs have often been investigated in isolation, their interaction receiving scant attention [48, 49]. This research represents a pioneering endeavor, unraveling the collective, influential role of these entities in the peritoneal metastasis of GC, furnishing a novel vantage point from which to comprehend GC metastasis.

Evolving treatment strategies for GC, especially those centered on mitigating metastasis, find in this study a new potential ally. The TGF-β/Smad2/3-LIF axis, as unveiled by our findings, proposes itself as a novel therapeutic nexus. Contrary to erstwhile strategies which targeted solitary molecules or pathways, a comprehensive approach, targeting the newly illuminated axis, may pave the way towards new therapeutic horizons for GC patients.

While this investigation propounds a novel theoretical foundation for understanding GC metastasis, it is not without limitations. Factors such as sample size might introduce elements that restrict the universal applicability of findings, and a deeper dive into the mechanisms underpinning the TGF-β/Smad2/3-LIF axis remains imperative.

### Supplementary information

Supplementary files

aj-checklist

## Data Availability

The data that supports the findings of this study are available on request from the corresponding author.
