# Supplementary material for: TGF-β-driven LIF expression influences neutrophil extracellular traps (NETs) and contributes to peritoneal metastasis in gastric cancer
Source: Cell Death Dis. 2024 Mar 15;15(3):218. doi: 10.1038/s41419-024-06594-w (PMC10943116; doi:10.1038/s41419-024-06594-w)
Supplement: Supplementary file 1 — Supplementary files [file 41419_2024_6594_MOESM1_ESM.pdf]

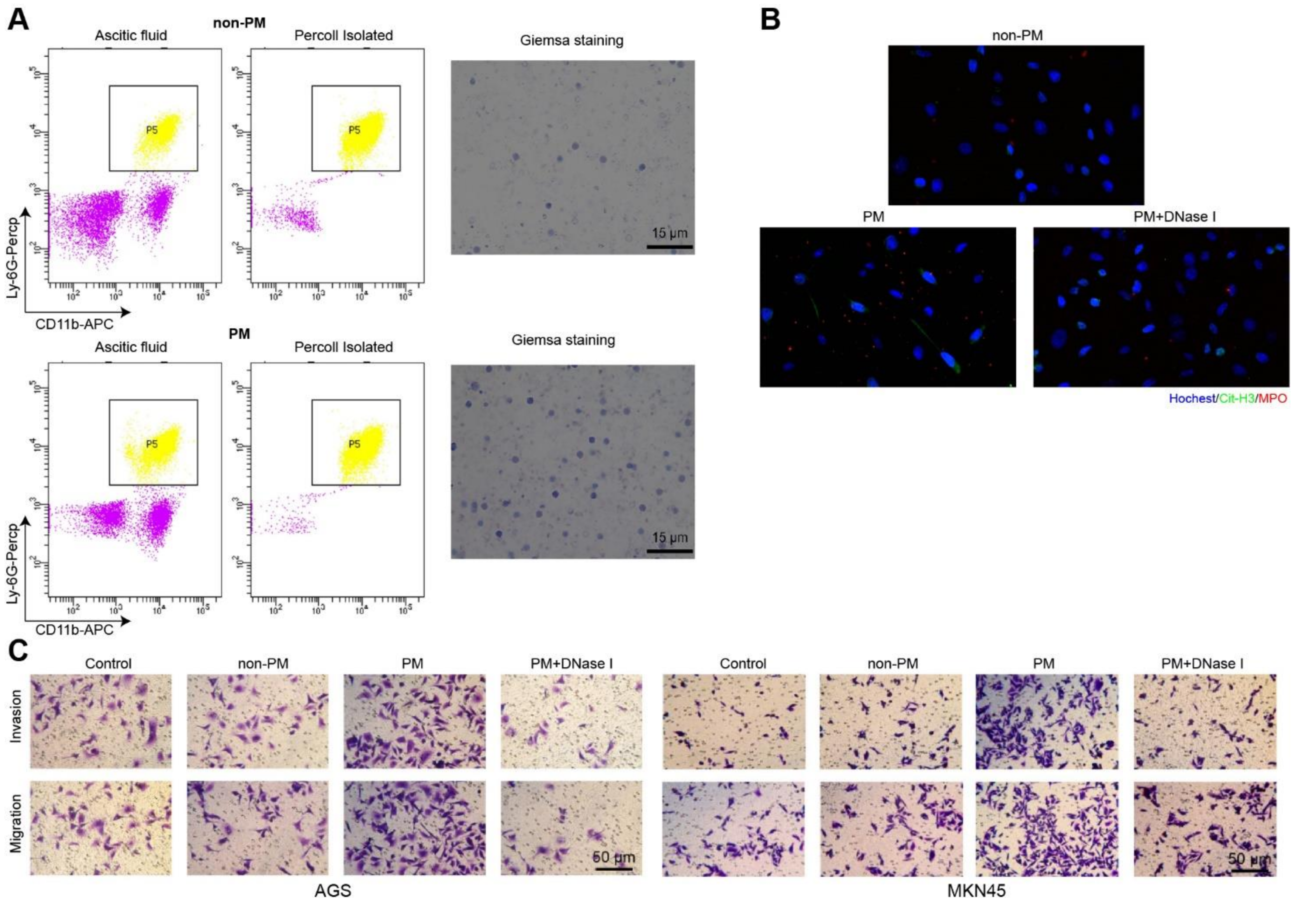

Fig. S1 Neutrophil NET formation affects GC cell proliferation, invasion, migration, and EMT in vitro.

Note: (A) Flow cytometry and Giemsa staining were used to verify the presence of neutrophils isolated from the ascites of non-PM group (n=12) and PM group (n=18) GC patients, with a scale bar of 50 μm; (B) Immunofluorescence co-staining was performed to assess the formation of NETs, with Cit-H3 labeled in green and MPO labeled in red, while the cell nuclei were labeled in blue; (C) Transwell assay was used to evaluate the migration and invasion of GC cells after co-culture. Cell experiments should be repeated at least three times.

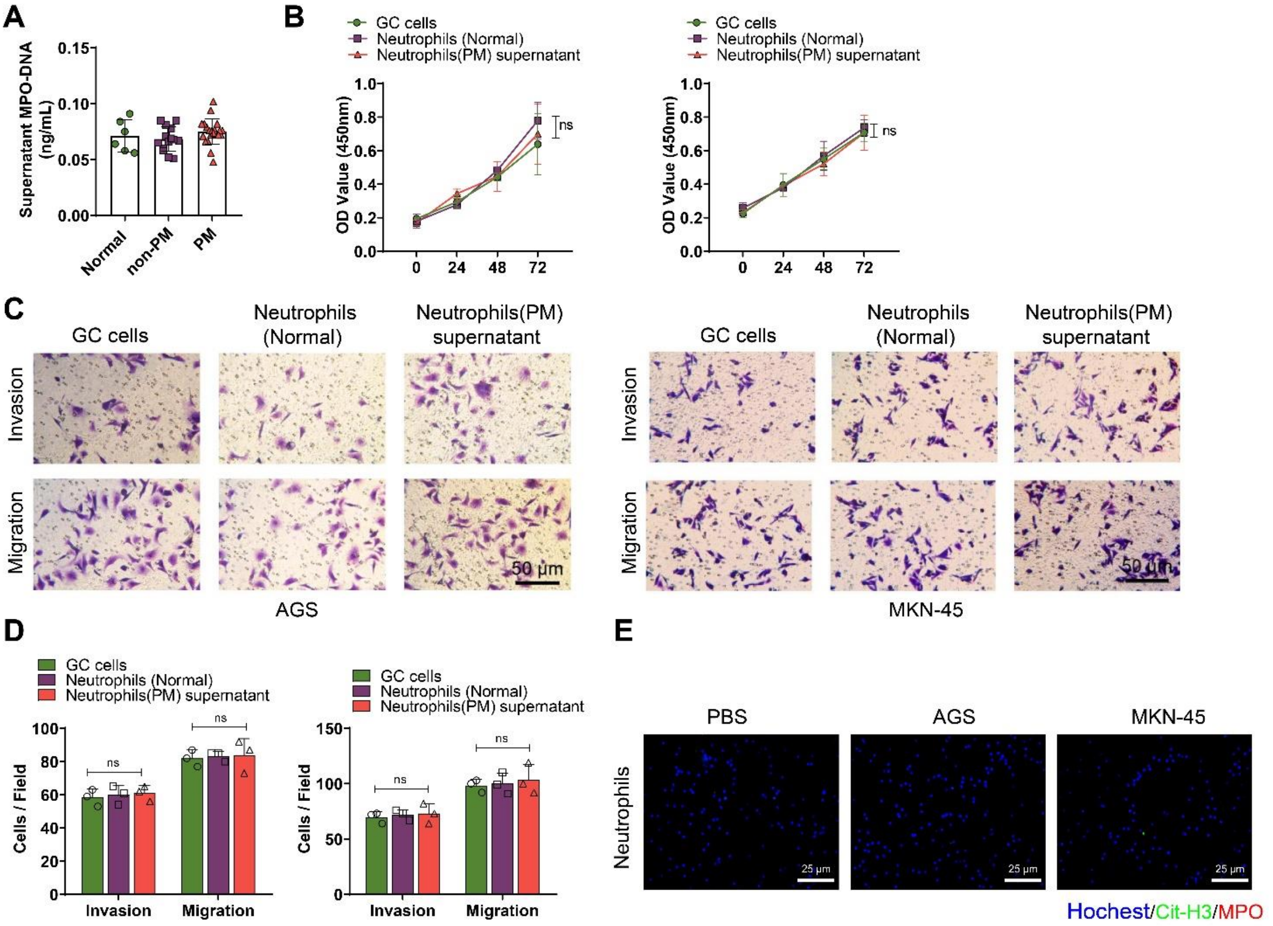

Fig. S2 The impact of NET-deficient neutrophils on the proliferation, invasion, and migration of GC cells.

Note: (A) ELISA detects the levels of MPO in the supernatant of neutrophil cultures from different sources;(B) CCK8 detects the proliferative ability of GC cells;(C) Transwell detects the migration and invasion of GC cells;(D) Transwell detects the migration and invasion ability of GC cells and presents the statistical data in a graph;(E) Immunofluorescence co-staining detects the levels of Cit-H3 and MPO in neutrophils and GC cells co-cultured, with nucleus labeled in blue, Cit-H3 labeled in green, and MPO labeled in red. Ns represents no significant difference, and the cell experiment should be repeated thrice.

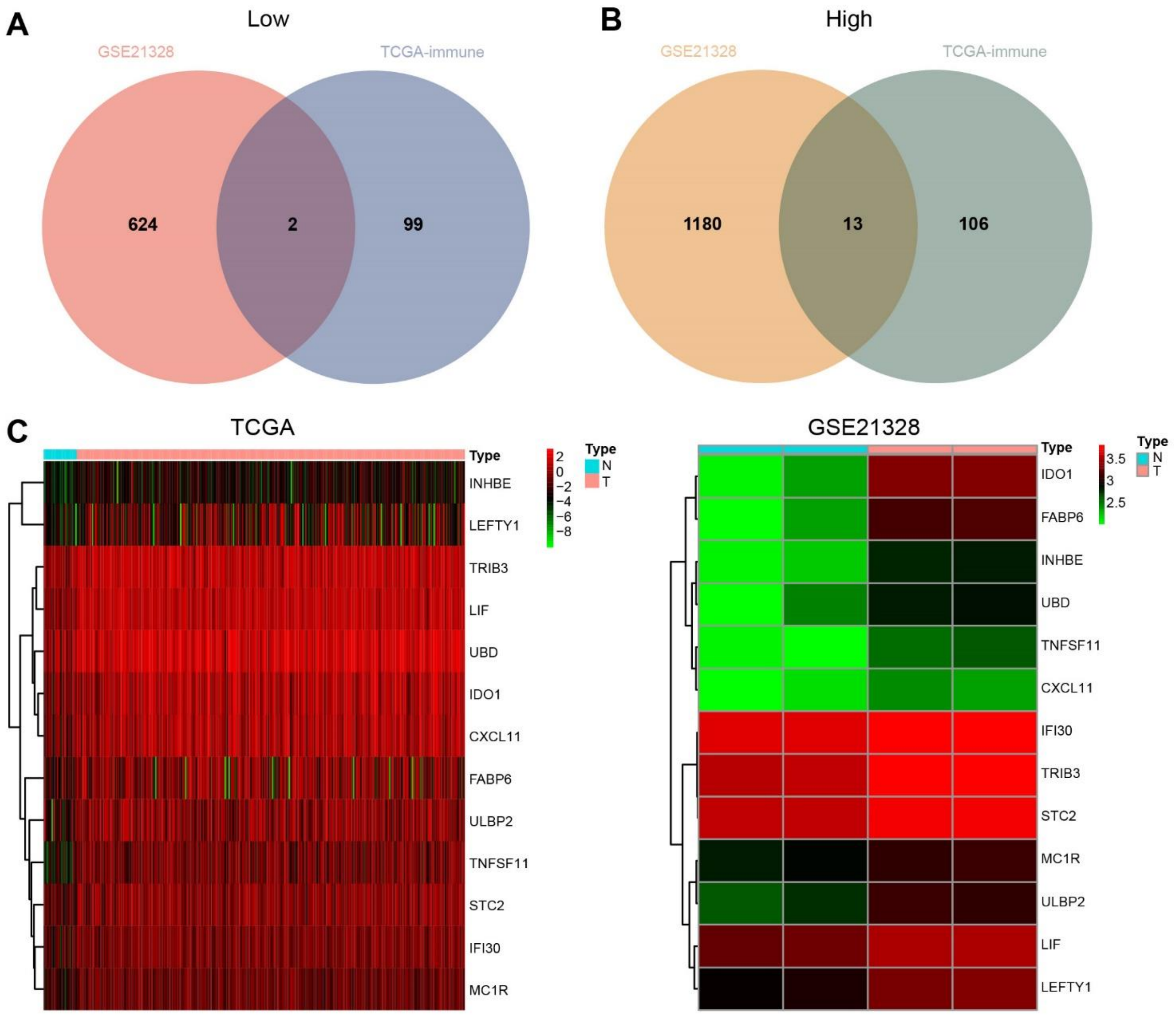

Fig. S3 Differential gene analysis of GEO database chip GSE21328 and TCGA database GC immune-related differential genes.

Note: (A) The intersection of the Venn diagram of downregulated genes in the GEO database GSE21328 (two samples from the highly metastatic GC cell line MKN-45-P and its parental cell line MKN-45) and the TCGA database GC immune-related downregulated genes (Normal: n=32, Tumor: n=327); (B) The intersection Venn diagram of downregulated genes in the GEO database GSE21328 (two samples from the highly metastatic GC cell line MKN-45-P and its parental cell line MKN-45) and the TCGA database GC immune-related upregulated genes (Normal: n=32, Tumor: n=327); (C) Expression heatmap of the 13 intersecting upregulated genes in the TCGA database and the GSE21328 chip dataset.

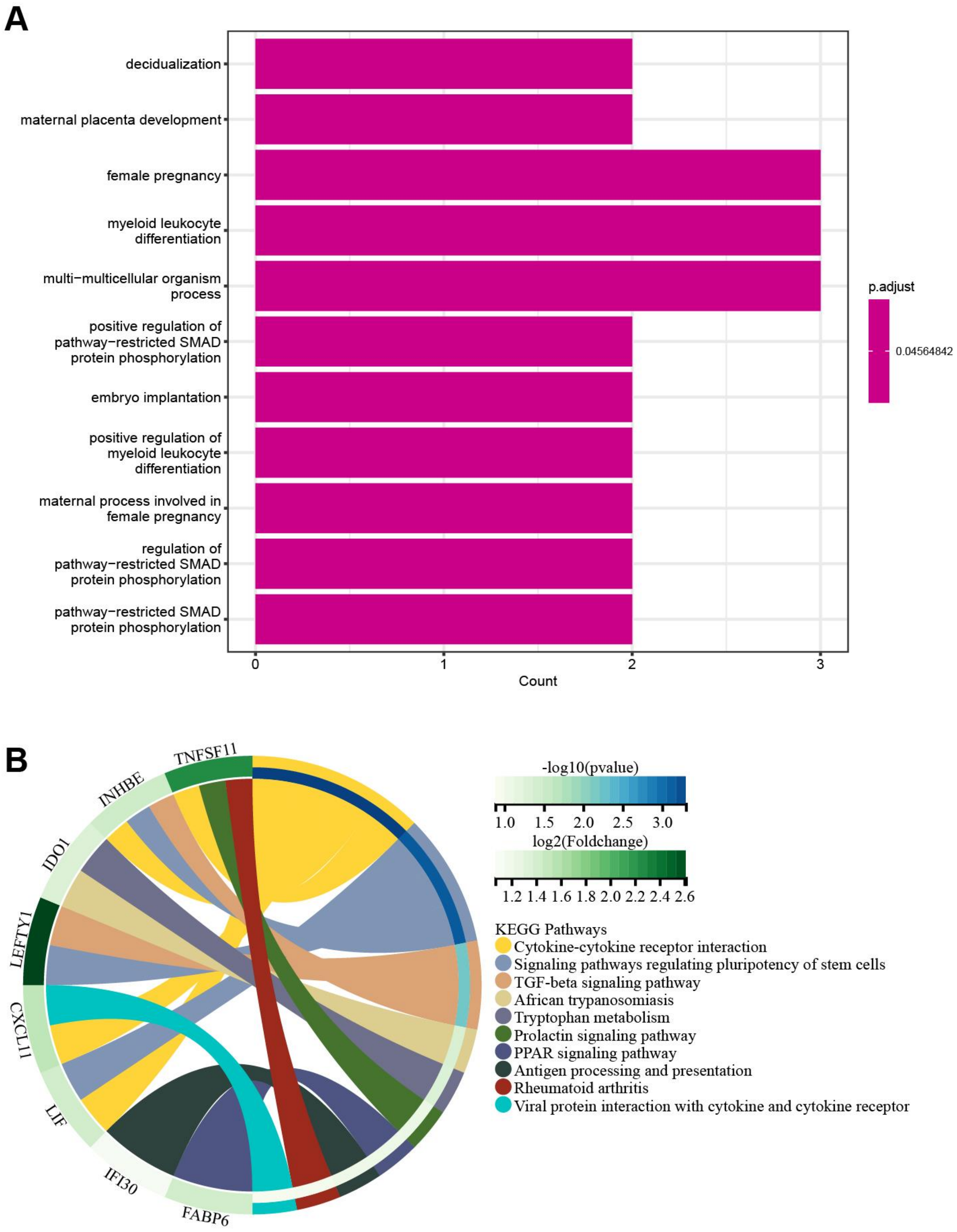

Fig. S4 Functional enrichment analysis of candidate target genes.
Note: (A) Bar chart of GO-BP functional analysis of 13 differentially upregulated intersection genes; (B) Circle diagram of KEGG functional enrichment analysis of 13 differentially upregulated intersection genes.

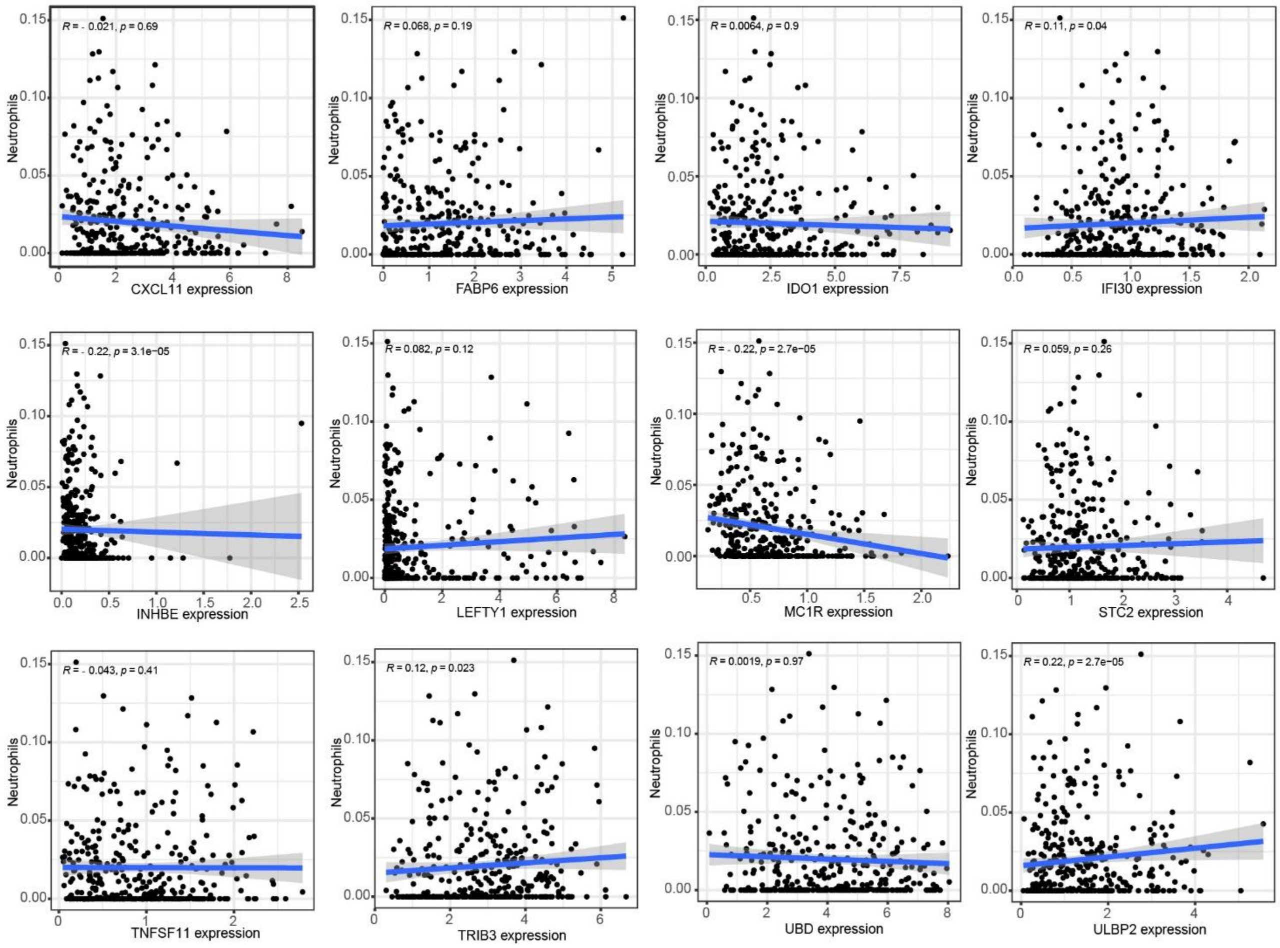

Fig. S5 Correlation analysis between candidate target genes and neutrophil infiltration. Note: We used CIBERSORT analysis to analyze the correlation between neutrophil infiltration and key genes in gastric cancer patients from the TCGA database.

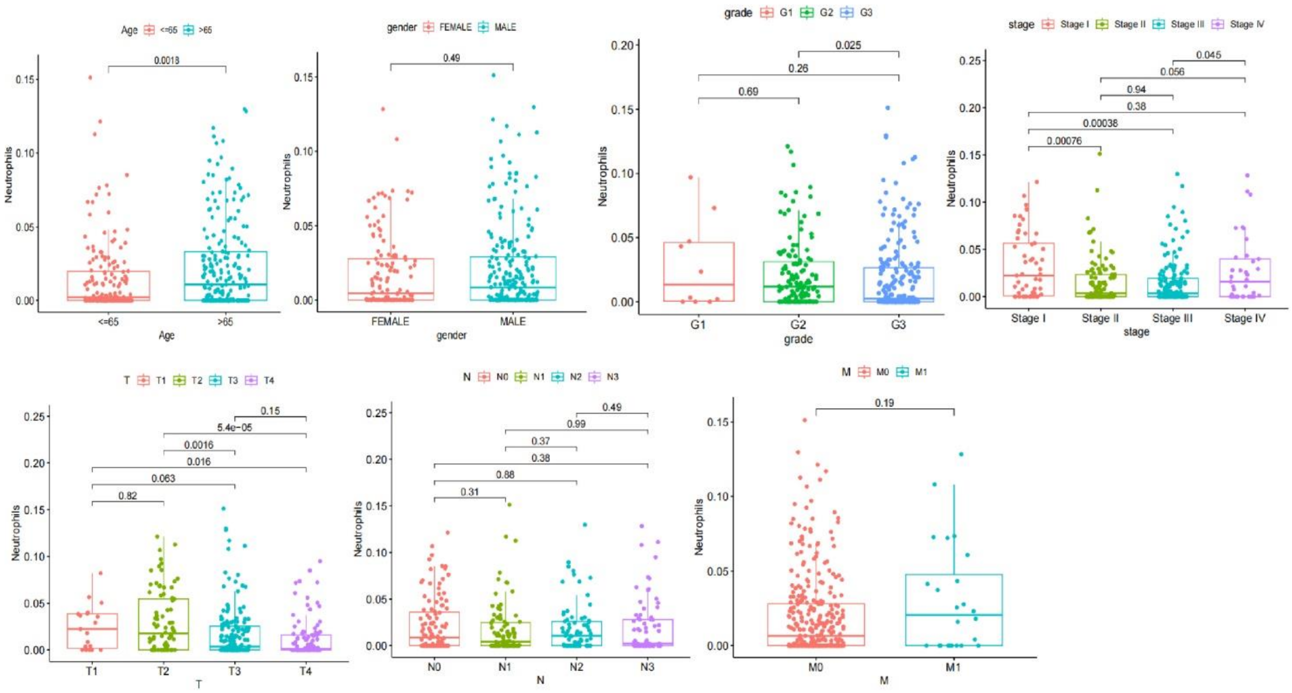

Fig. S6. Clinical correlation analysis of neutrophil infiltration.

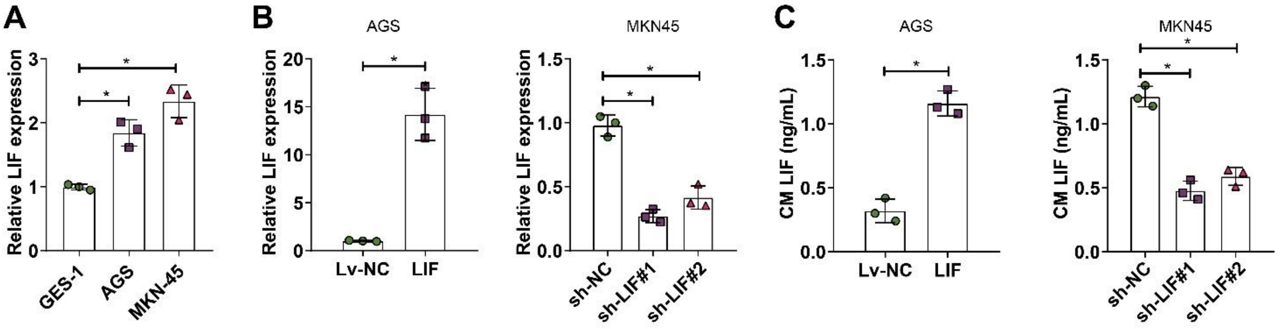

Fig. S7 Differential expression of LIF in GC cells.

Note: (A) RT-qPCR was used to detect the expression levels of LIF mRNA in GES-1 and GC cells;(B) RT-qPCR was used to detect the expression levels of LIF mRNA in GC cells after treatment with lentivirus;(C) ELISA was used to detect the levels of LIF in the culture medium of GC cells after treatment with lentivirus.
***P<0.05, cell experiments were repeated at least three times.**

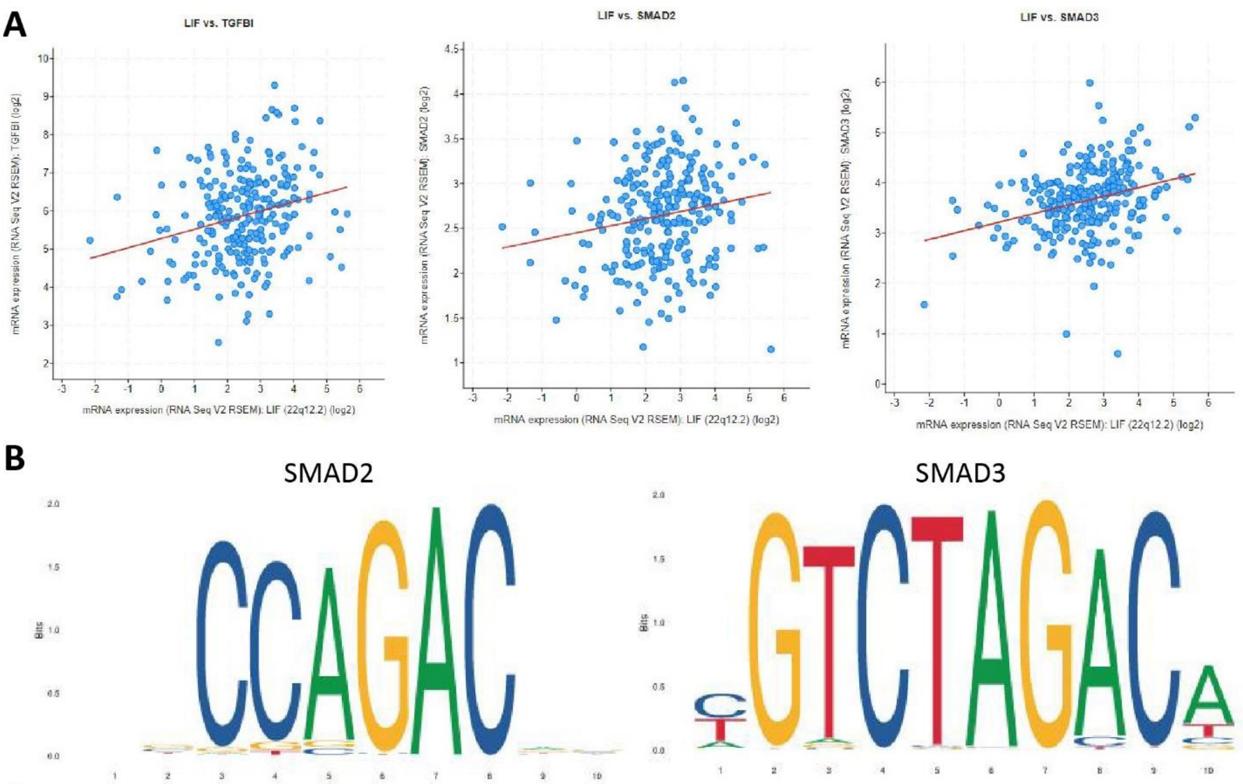

C

SMAD2

Matrix ID	Name	Score	Relative score	Sequence ID	Start	End	Strand	Predicted sequence	
MA1964.1	MA1964.1.SMAD2	12.14257	0.9776442462632178	LIF	565	574	-	ttccagacac	P1
MA1964.1	MA1964.1.SMAD2	7.09817	0.8733161178610563	LIF	878	887	-	gtcccgacat	P2
MA1964.1	MA1964.1.SMAD2	6.8995676	0.8692086329568164	LIF	1054	1063	-	ctcccgactc	P3

SMAD3

Matrix ID	Name	Score	Relative score	Sequence ID	Start	End	Strand	Predicted sequence	
MA0795.1	MA0795.1.SMAD3	5.59335	0.8146907353919992	LIF	879	888	-	tgtcccgaca	P2
MA0795.1	MA0795.1.SMAD3	5.2871084	0.810056329031904	LIF	566	575	-	cttccagaca	P1
MA0795.1	MA0795.1.SMAD3	-3.3917692	0.6787173566980721	LIF	1055	1064	-	tctcccgact	P3

Fig. S8 Predicted transcriptional regulation of LIF by the TGF-β/Smad signaling axis.
Note: (A) Correlation analysis of LIF and TGF-β, Smad2, and Smad3 in GC tissue, N=30; (B) Transcription factor Smad2 and Smad3 transcription regulatory site logo; (C) JASPAR predicted binding sites of Smad2/3 in the LIF promoter region.

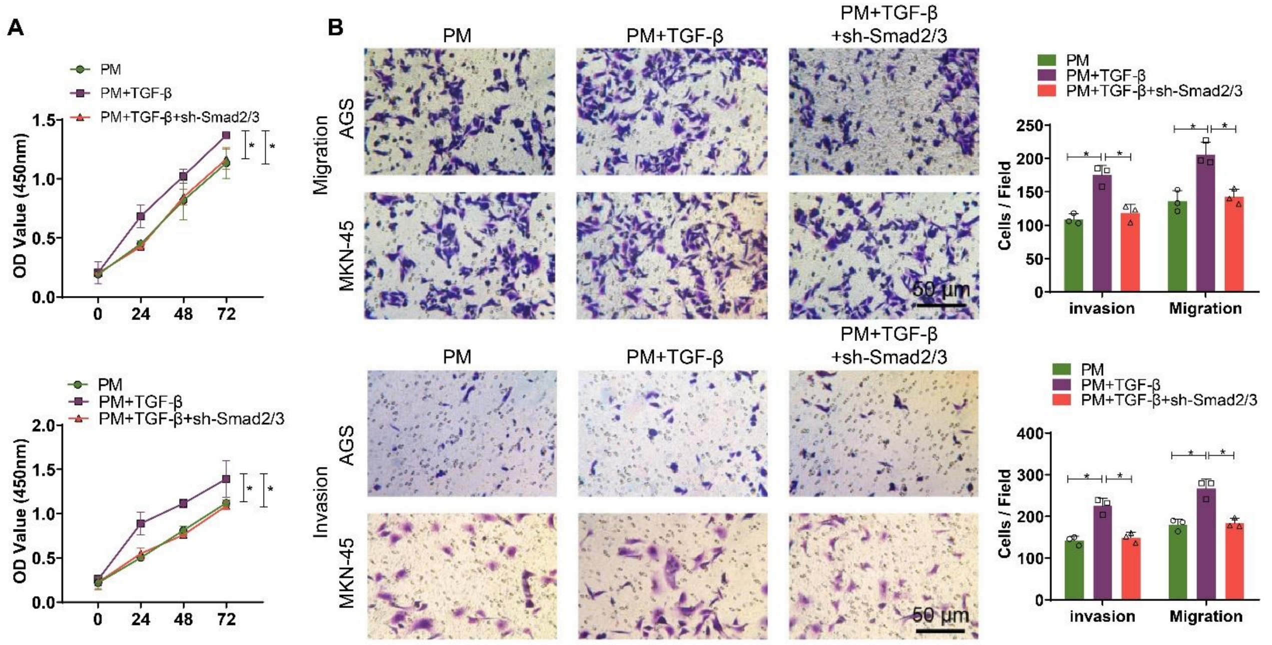

Fig. S9 The impact of the TGF-β/Smad/LIF signaling axis on GC cell proliferation, invasion, and migration.

Note: (A) After the knockdown of Smad2/3 by shRNA lentivirus for 3 h, GC cells were treated with 10 ng/mL TGF-β1, then co-cultured with neutrophils derived from peritoneal macrophages. The proliferation ability of GC cells was detected by CCK8 assay;(B) GC cells' migration and invasion ability were assessed using Transwell assay after co-culturing with neutrophils. *P<0.05, cell experiments were repeated at least three times.

Table S1 The clinicopathological characteristics of patients with GC

**Baseline characteristics**	**GC (n = 30)**
**Gender**	
**Male**	**17**
**Female**	**13**
**Age (years)**	
**< 65**	**14**
**≥ 65**	**16**
**Tumor size**	
**< 5 cm**	**16**
**≥ 5 cm**	**14**
**Tumor stage**	
**T1 + T2**	**12**
**T3 + T4**	**18**
**Histological grade**	
**Well/moderate**	**17**
**Poor/NS**	**13**
**Peritoneal metastasis**	
**Negative**	**12**
**Positive**	**18**
**Clinical stages**	
**I + II**	**13**
**III + IV**	**17**

Table S2 shRNA sequences

shRNA (sh-)	Sequence (5’-3’)
sh-NC	CCTAAGGTTAAGTCGCCCTCG
sh-LIF#1	GCAGTGCCAATGCCCTCTTTA
sh-LIF#2	GAACCAGATCAGGAGCCAACT
sh-Smad2	GCACTTGCTCTGAAATTTG
sh-Smad3	AATGGTGCGAGAAGGCGGTCA

Table S3 Primer sequences of ChIP-qPCR

Gene	Sequence (5'-3')
LIF-P1	Forward: GAAAACTGCCGGCATCTGAG<br>Reverse: GCCACACCCCTATATCTCACC
LIF-P2	Forward: TGGGATGCTGGGACGAAC<br>Reverse: TAGACGCTTTTCCAGGGCTC
LIF-P3	Forward: TGCGCTAGGTGAGATATAGGG<br>Reverse: CCCTGACTCCATGCCTTCTC

Table S4 The primer sequence for RT-qPCR

Gene	Sequence
LIF	Forward: 5’-CCAACGTGACGGACTTCCC-3’<br>Reverse: 5’-TACACGACTATGCGGTACAGC-3’
E-cadherin	Forward: 5’-ATTTTTCCCTCGACACCCGAT-3’<br>Reverse: 5’-TCCCAGGCGTAGACCAAGA-3’
Vimentin	Forward: 5’-TGCCGTTGAAGCTGCTAACTA-3’<br>Reverse: 5’-CCAGAGGGAGTGAATCCAGATTA-3’
Snail	Forward:5’-TGCCCTCAAGATGCACATCCGA-3’<br>Reverse:5’-GGGACAGGAGAAGGGCTTCTC-3’
Twist	Forward:5’-GCCAGGTACATCGACTTCCTCT-3’<br>Reverse:5’-TCCATCCTCCAGACCGAGAAGG-3’
N-cadherin	Forward:5’-CCTCCAGAGTTTACTGCCATGAC-3’<br>Reverse:5’-GTAGGATCTCCGCCACTGATTC-3’
GAPDH	Forward: 5’-CTGGGCTACACTGAGCACC-3’<br>Reverse: 5’-AAGTGGTCGTTGAGGGCAATG-3’

Table S5 KEGG and GO enrichment analysis of the 13 candidate target genes

ID	Description	GeneRatio	BgRatio	*p* value	*p*.adjust	*q* value	geneID	Count
GO:0046697	decidualization	13th Feb.	26/18723	0.000143	0.045648	0.028433034	LIF/STC2	2
GO:0001893	maternal placenta development	13th Feb.	35/18723	0.000261	0.045648	0.028433034	LIF/STC2	2
GO:0007565	female pregnancy	13th Mar.	193/18723	0.000286	0.045648	0.028433034	LIF/STC2/IDO1	3
GO:0002573	myeloid leukocyte differentiation	13th Mar.	208/18723	0.000356	0.045648	0.028433034	LIF/TNFSF11/UBD	3
GO:0044706	multi-multicellular organism process	13th Mar.	220/18723	0.00042	0.045648	0.028433034	LIF/STC2/IDO1	3
GO:0010862	positive regulation of pathway-restricted SMAD protein phosphorylation	13th Feb.	49/18723	0.000514	0.045648	0.028433034	INHBE/LEFTY1	2
GO:0007566	embryo implantation	13th Feb.	53/18723	0.000601	0.045648	0.028433034	LIF/STC2	2
GO:0002763	positive regulation of myeloid leukocyte differentiation	13th Feb.	58/18723	0.00072	0.045648	0.028433034	LIF/TNFSF11	2
GO:0060135	maternal process involved in female pregnancy	13th Feb.	62/18723	0.000822	0.045648	0.028433034	LIF/STC2	2
GO:0060393	regulation of pathway-restricted SMAD protein phosphorylation	13th Feb.	62/18723	0.000822	0.045648	0.028433034	INHBE/LEFTY1	2
GO:0060389	pathway-restricted SMAD protein phosphorylation	13th Feb.	65/18723	0.000903	0.045648	0.028433034	INHBE/LEFTY1	2
GO:0060395	SMAD protein signal transduction	13th Feb.	82/18723	0.001432	0.066367	0.041338001	INHBE/LEFTY1	2

**GO:0051098**	**regulation of binding**	**13th Mar.**	**363/18723**	**0.001789**	**0.076513**	**0.047657946**	**LIF/TNFSF11/TRIB3**	**3**

Original western blots

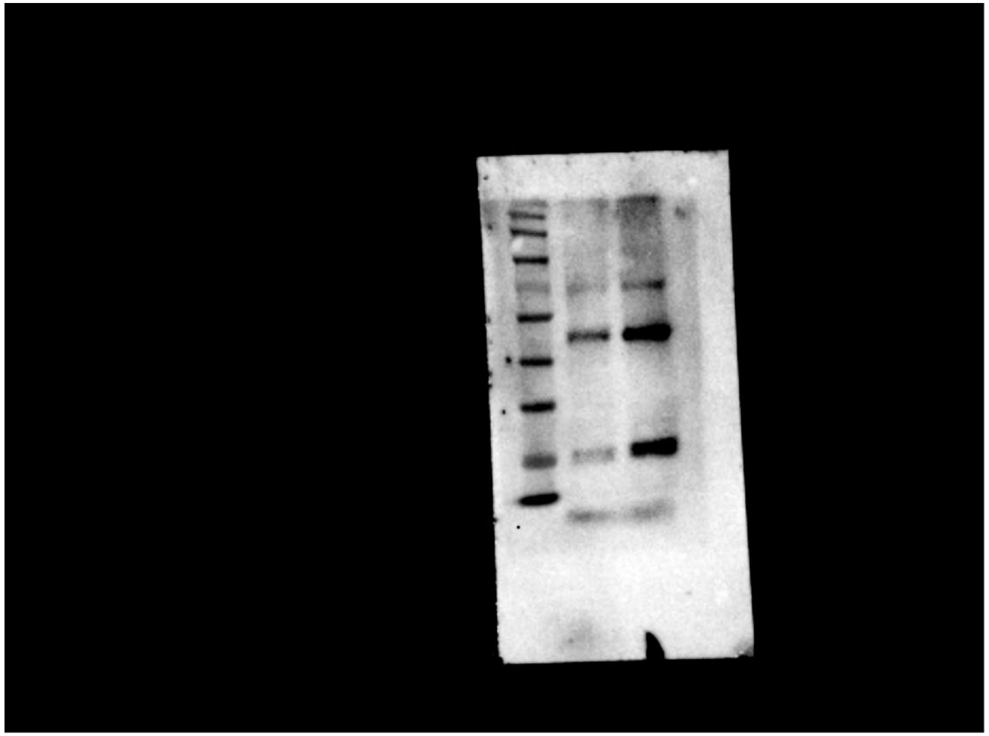

Figure5C-1-1

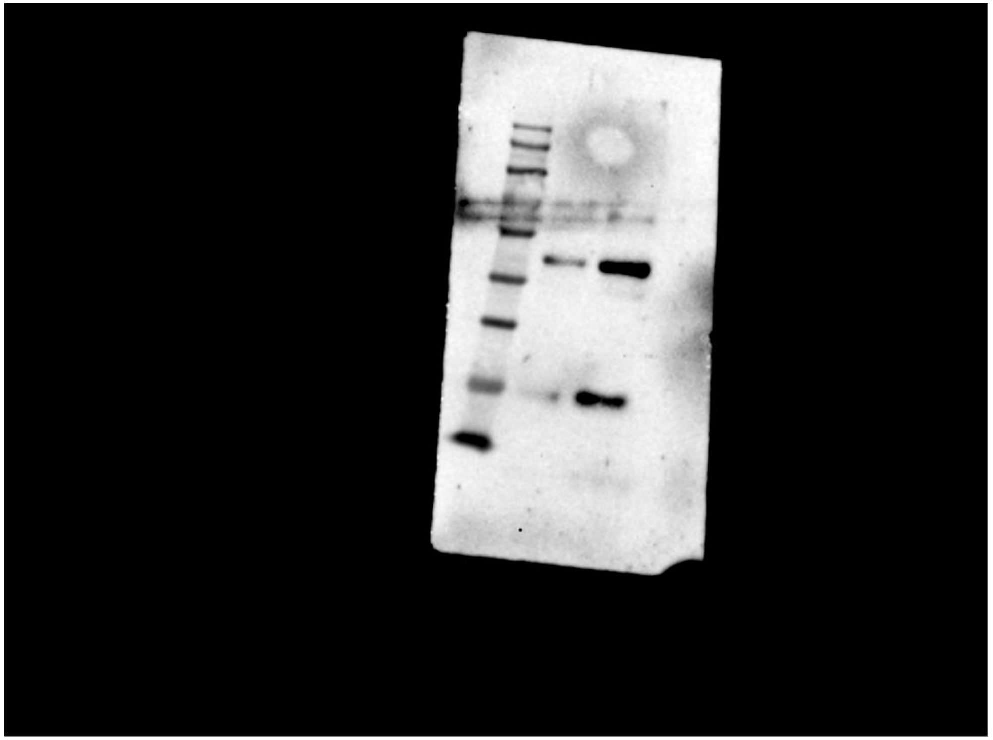

Figure5C-1-2

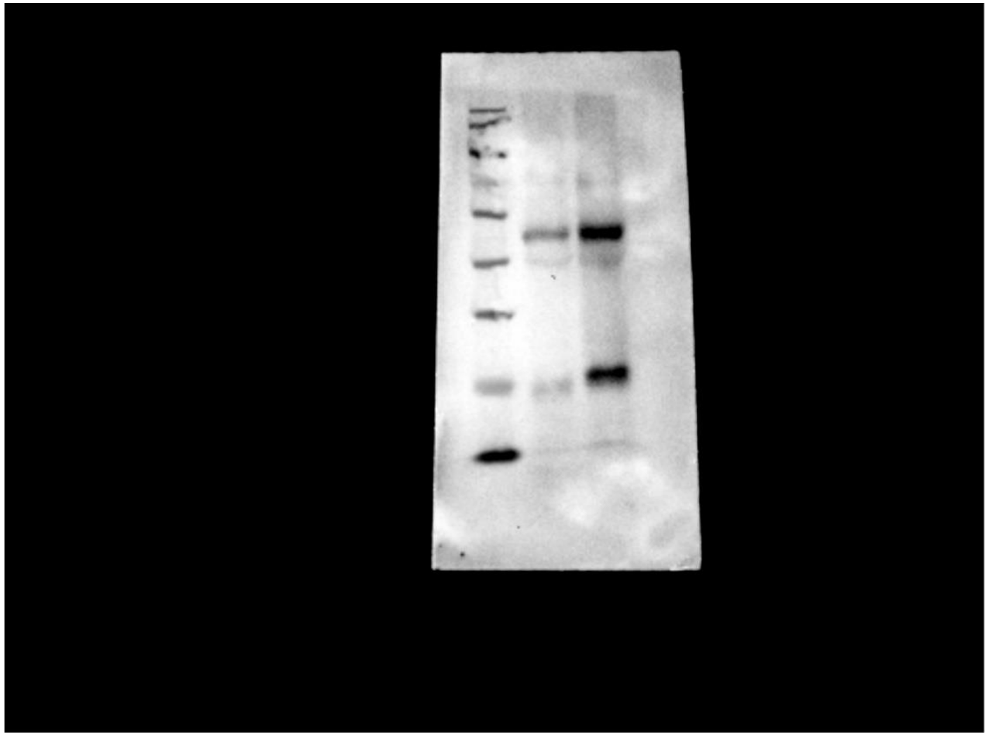

Figure5C-1-3

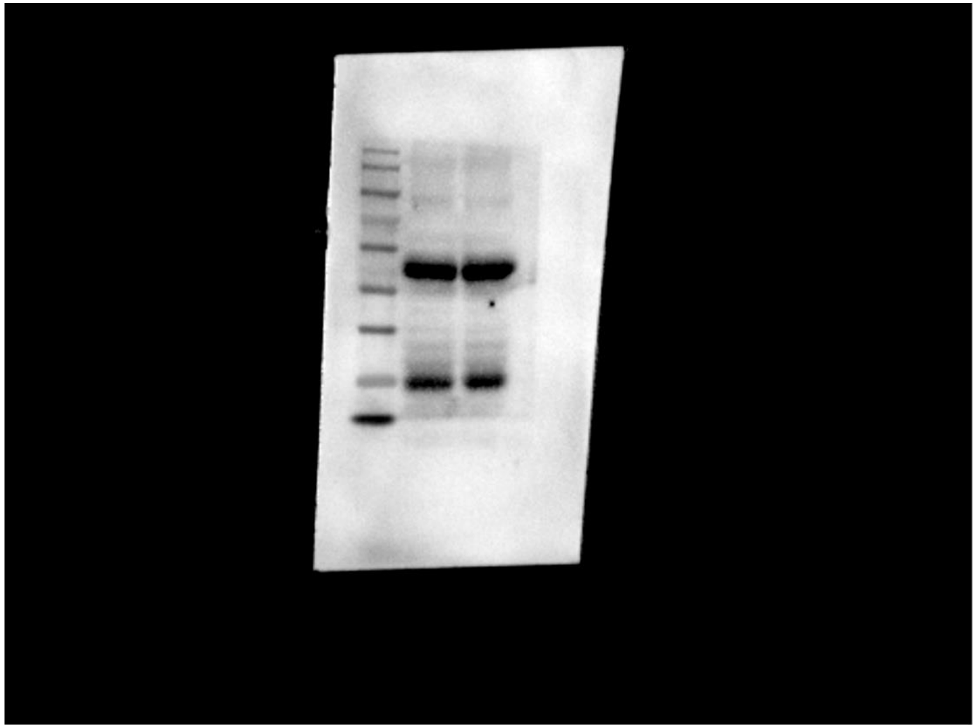

Figure5C-1-4

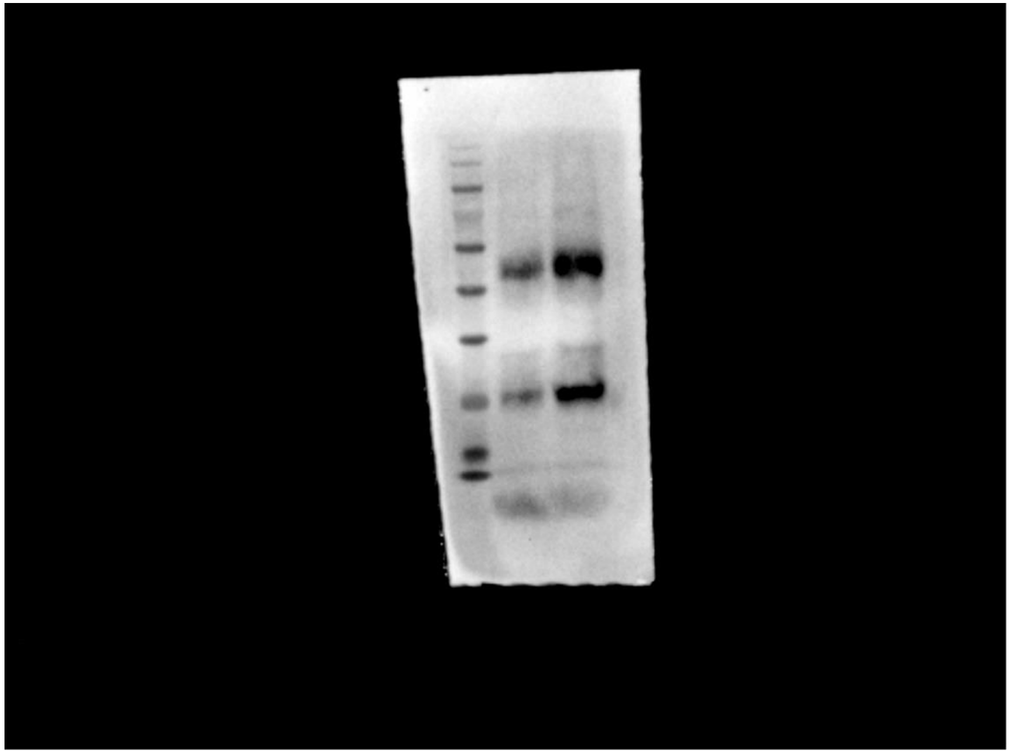

Figure5C-2-1

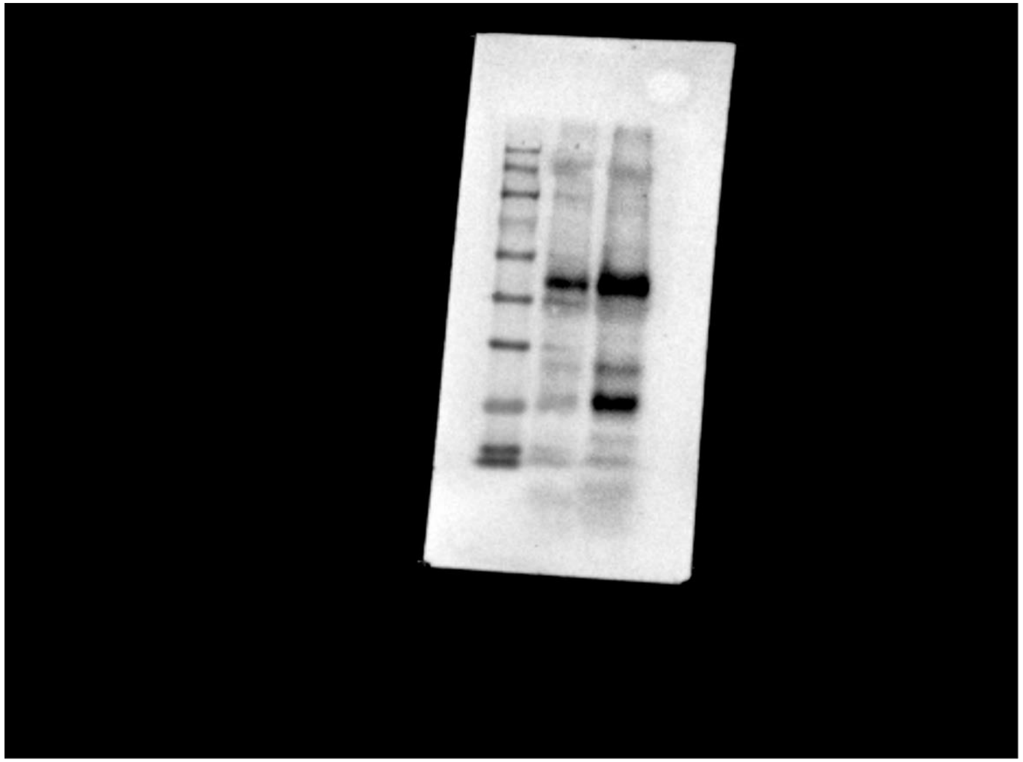

Figure5C-2-2

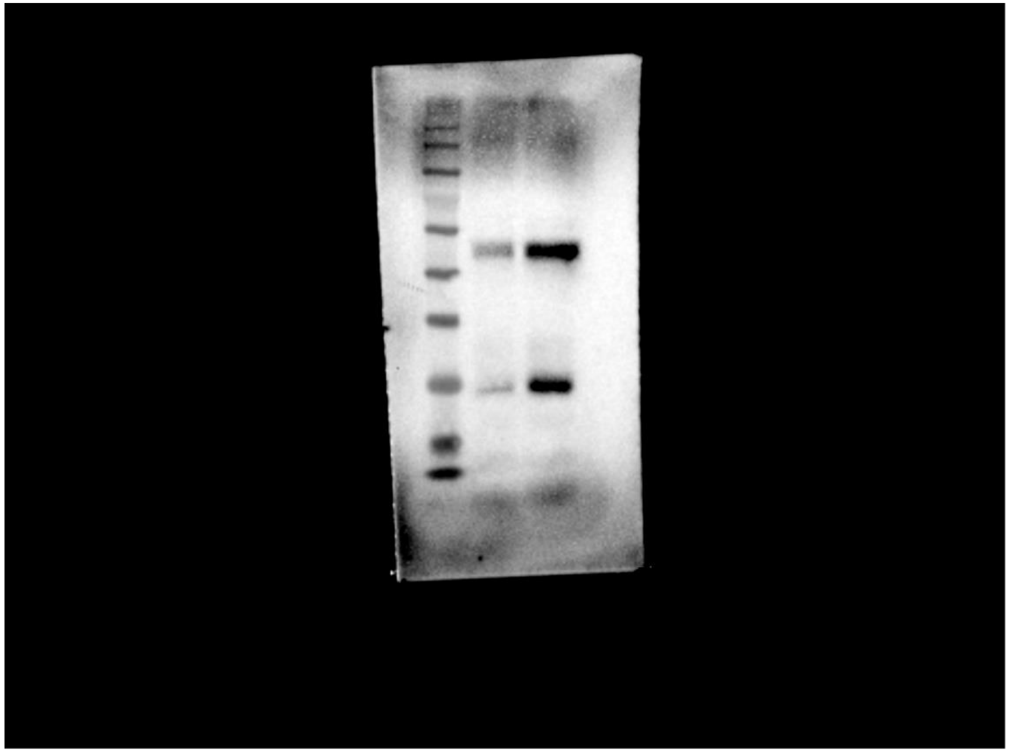

Figure5C-2-3

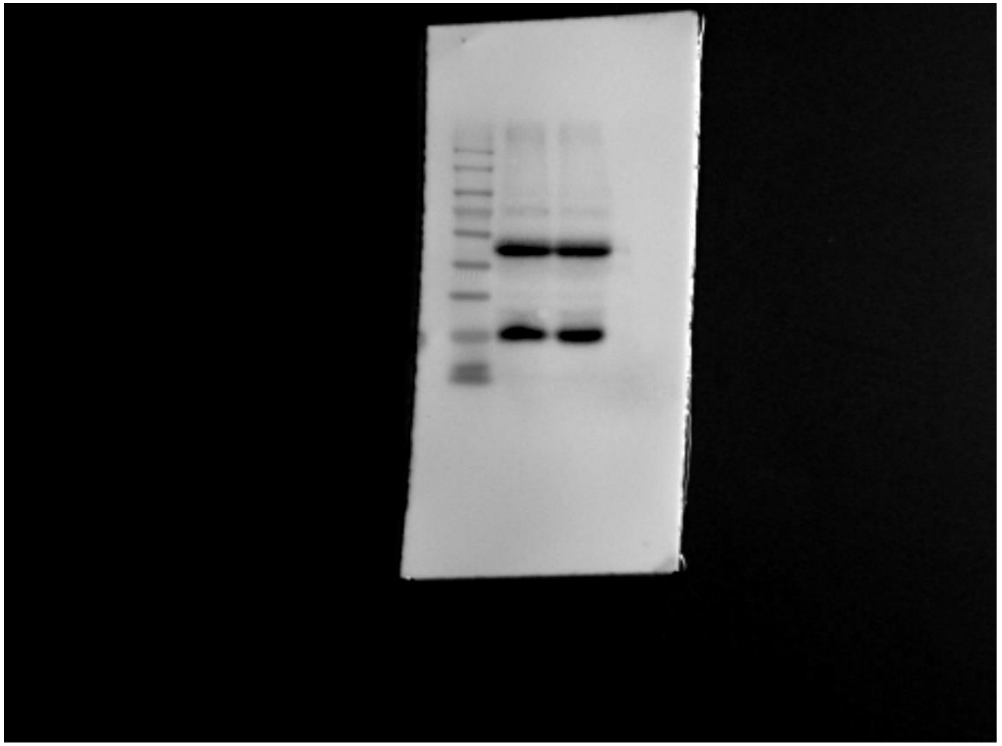

Figure5C-2-4
